# Imaging PD-L1 in the brain—Journey from the lab to the clinic

**DOI:** 10.1093/neuonc/noae190

**Published:** 2024-10-28

**Authors:** Dawoud Dar, Magdalena Rodak, Chiara Da Pieve, Izabela Gorczewska, Gitanjali Sharma, Ewa Chmielik, Marcin Niedbala, Pawel Bzowski, Andrea d’Amico, Barbara Bobek-Billewicz, Elzbieta Nowicka, Rafal Tarnawski, Wojciech Kaspera, Gabriela Kramer-Marek

**Affiliations:** Department of Radiotherapy and Imaging, Institute of Cancer Research, London, UK; Department of Radiopharmacy and Preclinical PET Imaging, Maria Sklodowska-Curie National Research Institute of Oncology, Gliwice, Poland; Department of Radiotherapy and Imaging, Institute of Cancer Research, London, UK; Department of Nuclear Medicine and Endocrine Oncology, Maria Sklodowska-Curie National Research Institute of Oncology, Gliwice, Poland; Department of Radiotherapy and Imaging, Institute of Cancer Research, London, UK; Department of Tumor Pathology, Maria Sklodowska-Curie National Research Institute of Oncology, Gliwice, Poland; Department of Neurosurgery, Medical University of Silesia, Katowice, Poland; Department of Nuclear Medicine and Endocrine Oncology, Maria Sklodowska-Curie National Research Institute of Oncology, Gliwice, Poland; Department of Nuclear Medicine and Endocrine Oncology, Maria Sklodowska-Curie National Research Institute of Oncology, Gliwice, Poland; Department of Radiology and Imaging Diagnostic, Maria Sklodowska-Curie National Research Institute of Oncology, Gliwice, Poland; Department of IIIrd Radiotherapy and Chemotherapy, Maria Sklodowska-Curie National Research Institute of Oncology, Gliwice, Poland; Department of IIIrd Radiotherapy and Chemotherapy, Maria Sklodowska-Curie National Research Institute of Oncology, Gliwice, Poland; Department of Neurosurgery, Medical University of Silesia, Katowice, Poland; Department of Radiopharmacy and Preclinical PET Imaging, Maria Sklodowska-Curie National Research Institute of Oncology, Gliwice, Poland; Department of Radiotherapy and Imaging, Institute of Cancer Research, London, UK

**Keywords:** glioblastoma, immuno-PET, immunotherapy, nuclear medicine, translational studies

## Abstract

**Background:**

Immune checkpoint inhibitors (ICPIs) have proven to restore adaptive anti-tumor immunity in many cancers; however, no noteworthy therapeutic schedule has been established for patients with glioblastoma (GBM). High programmed death-ligand 1 (PD-L1) expression is associated with immunosuppressive and aggressive phenotypes in GBM. Presently, there is no standardized protocol for assessing PD-L1 expression levels to select patients and monitor their response to ICPI therapy. The aim of this study was to investigate the use of ^89^Zr-DFO-Atezolizumab to image the spatio-temporal distribution of PD-L1 in preclinical mouse models and in patients with newly diagnosed GBM treated with/without neoadjuvant Pembrolizumab.

**Methods:**

The immunoreactivity, binding affinity, and specificity of ^89^Zr-DFO-Atezolizumab were confirmed in vitro. Mice-bearing orthotopic GBM tumors or patients with newly diagnosed GBM treated with/without Pembrolizumab were intravenously injected with ^89^Zr-DFO-Atezolizumab, and PET/CT images were acquired 24, 48, and 72 hours in mice and at 48 and 72 post-injection in patients. Radioconjugate uptake was quantified in the tumor and healthy tissues. Ex vivo immunohistochemistry (IHC) and immunophenotyping were performed on mouse tumor samples or resected human tumors.

**Results:**

^89^Zr-DFO-Atezolizumab was prepared with high radiochemical purity (RCP > 99%). In vitro cell-associated radioactivity of ^89^Zr-DFO-Atezolizumab corroborated cell line PD-L1 expression. PD-L1 in mouse GBM tumors was detected with high specificity using ^89^Zr-DFO-Atezolizumab and radioconjugate uptake correlated with IHC. Patients experienced no ^89^Zr-DFO-Atezolizumab-related side effects. High ^89^Zr-DFO-Atezolizumab uptake was observed in patient tumors at 48 hours post-injection, however, the uptake varied between patients treated with/without Pembrolizumab.

**Conclusions:**

^89^Zr-DFO-Atezolizumab can visualize distinct PD-L1 expression levels with high specificity in preclinical mouse models and in patients with GBM, whilst complementing ex vivo analysis.

**Key Points**

- ^89^Zr-DFO-Atezolizumab can accurately and noninvasively detect spatial and temporal changes in PD-L1 expression in GBM.
- ^89^Zr-DFO-Atezolizumab can track PD-L1 changes within the tumor and lymphoid organs, indicating systemic changes induced by neoadjuvant Pembrolizumab.

**Importance of the Study**

PD-L1 is a highly dynamic biomarker and exhibits heterogeneity within GBM tumors, which cannot be captured with the local and static information provided by IHC. Furthermore, there are no standardized scoring criteria and staining protocol for PD-L1 in GBM which leads to discrepancies in results between patients. The assessment of PD-L1 is crucial for monitoring patient response to PD-1/PD-L1 blockade therapies. Our data marks the first clinical use of ^89^Zr-DFO-Atezolizumab, providing a novel diagnostic tool to evaluate PD-L1 expression in GBM. This method enables precise and quantitative assessment of PD-L1 across the entire tumor and its distribution in the body. Importantly, our initial findings indicate that immuno-PET with ^89^Zr-DFO-Atezolizumab can effectively monitor dynamic PD-L1 changes within the tumor microenvironment and lymphoid organs, reflecting systemic immune modulation induced by neoadjuvant Pembrolizumab.

Our understanding of molecular and genetic alterations that drive glioblastoma (GBM) is constantly widening. However, no noteworthy effective therapy for GBM has been developed yet. Despite rigorous treatment regimens consisting of maximal surgical resection followed by adjuvant chemo-radiotherapy, the median overall survival (OS) of patients diagnosed with GBM is below 15 months.^1^ This highlights an unmet clinical need for new therapeutic strategies for GBM. Nowadays, it is well recognized that immune checkpoint inhibitors (ICPIs) targeting the programmed death receptor 1 (PD-1)/programmed death-ligand 1 (PD-L1) axis can restore previously suppressed T cell anti-tumor responses and significantly improve survival in many cancers.^2^ Nevertheless, their role in GBM still remains unclear. This is partly attributed to the heterogeneous and immunologically cold microenvironment of these tumors, which are characterized by elevated levels of tumor-associated macrophages (TAMs) and a low number of cytotoxic T lymphocytes (CTLs) with a predominantly exhausted-like phenotype.^3^ These complex features of GBM and its tumor microenvironment (TME) suggest that each patient may require a treatment regimen tailored individually to the unique characteristics of the tumor.

Several studies have shown that high PD-L1 expression in GBM is associated with invasiveness, immuno-resistance, and an overall poor prognosis.^4–8^ Furthermore, PD-L1 was found to be significantly upregulated in the more aggressive and multitherapy-resistant mesenchymal subtype of GBM amongst other transcriptional subtypes. Nevertheless, most clinical trials targeting PD-1/PD-L1 have failed to demonstrate a survival benefit in GBM patients. The Checkmate 143 clinical trial (NCT02017717) reported comparable median OS between nivolumab-treated and bevacizumab-treated control patients.^9^ Similarly, in the Checkmate 498 study (NCT02617589), combined PD-1 blockade and radiotherapy in newly diagnosed GBM patients with unmethylated MGMT promoter did not improve survival.^10^ Lastly, nivolumab in combination with temozolomide (TMZ) and radiotherapy was found not to be superior to TMZ, radiotherapy, and placebo in newly diagnosed GBM patients with methylated MGMT promoter.^11^

Interestingly, *Cloughesy* et al. demonstrated that compared to adjuvant PD-1 blockade, neoadjuvant PD-1 blockade with Pembrolizumab improved OS, enriched interferon-related genes, and increased T cell signatures in patients with recurrent GBM.^12^ Within the neoadjuvant group, a focal upregulation of PD-L1 induced by a release of interferon-γ (IFN-γ) from infiltrating CTLs suggested the need for an accurate diagnostic determination of PD-L1 to allow more effective patient stratification and monitoring of anti-PD-1 response.

Although immunohistochemistry (IHC) of biopsy specimens has been approved by the FDA as a companion or complementary diagnostic test for PD-L1 expression levels in certain cancers (e.g. NSCLC, HNSCC, and TNBC), alternative methods are still needed to overcome the pitfalls of the local and static information that is provided from small snapshots of resected tumor tissue.

Lately, several groups have shown that immuno-PET imaging using radiolabeled monoclonal antibodies (mAbs), antibody fragments and affibodies can characterize the spatial and temporal heterogeneity of immune system target molecules, highlighting the potential of imaging to guide personalized combinations of immunotherapy with local and/or systemic therapies.^13,14^*Bensch* et al. showed in a cohort of patients with NSCLC, TNBC, and metastatic bladder cancer that accumulation of ^89^Zr-Atezolizumab (anti-PD-L1) corresponds to PD-L1 IHC staining in inflammation sites and normal lymphoid tissues (NCT02453984 and NCT02478099).^15^ Higher tumor uptake of ^89^Zr-Durvalumab (anti-PD-L1) was found in NSCLC patients who responded to Durvalumab treatment, but the radioconjugate accumulation did not correlate with tumor PD-L1 IHC.^16^ Furthermore, ^89^Zr-Pembrolizumab (anti-PD-1) PET imaging of metastatic melanoma and NSCLC patients (NCT02760225) showed high and specific tracer accumulation in extracerebral tumor lesions, brain lesions, and lymph node metastases. Most importantly, all major sites where T cells can be found including normal lymphoid tissues and inflammation sites were also visualized.^17^

Herein, we present the results of the first immuno-PET imaging studies using ^89^Zr-DFO-Atezolizumab in patients with newly diagnosed GBM treated with/without neoadjuvant Pembrolizumab (NCT05235737). This is of particular importance as it has been previously demonstrated that neoadjuvant anti-PD-1 blockade improves survival outcomes in patients with recurrent GBM.^18^

Initially, ^89^Zr-DFO-Atezolizumab was tested in vitro to evaluate its affinity and specificity for PD-L1. Radioconjugate uptake was then imaged and measured in vivo using orthotopic human and murine GBM mouse models with varied PD-L1 status.

Finally, the whole-body PET scans of 8 GBM patients were performed. The primary objective of the clinical study was to determine the tumor uptake and whole-body biodistribution of ^89^Zr-DFO-Atezolizumab. The secondary objectives were: (1) to non-invasively characterize the spatial and temporal heterogeneity of PD-L1; and (2) to identify the relationship between ^89^Zr-DFO-Atezolizumab uptake, PD-L1 IHC staining, and tumor-infiltrating immune cell populations.

## Preclinical Methods

### Cell Culture

Murine GBM cell lines GL261 and GL261_PDL1KO_ were provided by Prof. Karl H. Plate (Goethe University) and were cultured in Dulbecco’s Modified Essential Medium (DMEM) supplemented with 10% fetal bovine serum (FBS; Gibco, Thermo Fisher Scientific). The human GCGR-E55 and the murine BL6-NPE-IE GBM cell lines were provided by Prof. Steven Pollard (University of Edinburgh) and cultured as described in Supplementary Materials.^19^ Human GBM cell line, U87-MGvIII, was provided by Dr Frank Furnari (Ludwig Cancer Research) and cultured in DMEM supplemented with 10% FBS and 400 µg/mL of selective antibiotic (Geneticin, G418 Sulfate, Thermo Fisher Scientific). All cells were maintained in a humidified chamber at 37 °C and supplied with 5% CO_2_. Polymerase chain reaction was regularly performed to verify negative mycoplasma status and short tandem repeat profiling was used to authenticate cell line identity (Surrey Diagnostics).

### Mouse Models

The detailed methods are described in Supplementary Materials. Briefly, all experiments were performed in accordance with the license issued under the UK Animals (Scientific Procedures) Act 1986, the UK National Cancer Research Institute Guidelines for Animal Welfare in Cancer Research^20^ and the ARRIVE guidelines for reporting animal research.^21^ The studies were conducted under the Project Licence PP3472375, approved by the UK Home Office and by the local ethical review committee. Female athymic nude mice (crl:NU(NCR)-Foxn1^nu^) and female C57BL/6J (6–8 weeks) were stereotactically injected with U87-MGvIII, GL261, or GL261_PDL1KO_ cells (1×10^5^) in 2 μL phosphate-buffered saline (PBS). Tumor growth was monitored with the 1T M3™ MRI system (Aspect Imaging). PET studies were performed once the brain tumors reached ~25 mm^3^.

### Preparation of IR700-Atezolizumab

The conjugation of IRDye700DX NHS ester (Rakuten Medical) further called IR700, to Atezolizumab (Tecentriq, 1200 mg/20 mL, Roche) is described in detail in the Supplementary Materials.

### Preparation of ^89^Zr-DFO-Atezolizumab

The conjugation of deferoxamine-maleimide (DFO-mal, Macrocyclics) to Atezolizumab (Tecentriq), ^89^Zr-radiolabeling procedures of ^89^Zr-DFO-Atezolizumab are described in detail in the Supplementary Materials. The radiochemical purity (RCP) was assessed by ITLC using ITLC-SG strips (Agilent Technologies). The strips were analyzed using a Scan-RAM Radio TLC Scanner and Analyzer (LabLogic) and the RCP was consistently >99%.

### Flow Cytometry

The experimental details are given in the Supplementary Materials. Briefly, GL261, GL261_PD-L1KO_, BL6-NPE-IE, GCGR-E55, and U87-MGvIII cells pretreated with 20 ng/mL of either human recombinant IFN-γ (Thermo Fisher Scientific) or mouse recombinant IFN-γ (Merck KGaA) were collected. To assess PD-L1 expression, cells were stained with PE-conjugated anti-mouse PD-L1 mAb (Clone: 10F.9G2, 124308, Biolegend), PE-Cyanine-7-conjugated anti-human PD-L1 mAb (Clone: MIH1, 25-5983-42, Invitrogen) or PBS (control) at 4 °C for 40 minutes. Flow cytometry was performed using the FACSymphony A5 Cell Analyzer (BD Biosciences).

### Tumor and Immune Cells Isolation

For multicolor flow cytometry, GL261 tumor-bearing brains were mechanically and enzymatically dissociated using the Brain Tumor Dissociation Kit (130-095-942, Miltenyi Biotec) and the gentle MACS^TM^ Octo Dissociator with heaters (130-096-427, Miltenyi Biotec). A single-cell suspension was incubated with either a T cell or myeloid cell marker panel of fluorescent primary antibodies for 1 hour at 4 °C. All experimental details with the list of fluorescently labeled primary antibodies are provided in the Supplementary Materials.

### Cellular Accumulation of IR700-Atezolizumab

The experimental details about IR700-Atezolizumab (1 μM) uptake are given in the Supporting Materials.

### Evans Blue Blood Brain Barrier Permeability Assay

C57BL/6J mice-bearing GL261 brain tumors were intravenously injected with 100 μL of 10 mg/ml Evans Blue (E2129, Merck KGaA). Brains were excised 45 minutes post-injection, followed by sectioning using a brain matrix (4 mm slices). The entire brain and individual sections were photographed, and Evans Blue fluorescence was imaged using the IVIS Spectrum/CT system (PerkinElmer) with 640/680 nm ex/em filters.

### ^89^Zr-DFO-Atezolizumab In Vitro Studies

The stability of ^89^Zr-DFO-Atezolizumab was assessed by incubating the purified product (ca. 3 MBq) in mouse serum (500 μL, Sigma Aldrich) in a thermo shaker (Grant-Bio PHMT, Thermo Fisher Scientific) at 37 °C (250 rpm) for up to 4 days. Aliquots were taken at different time points and analyzed by ITLC using ITLC-SG strips (*n* = 3).

The target-binding fraction was assessed following the method described in the literature^22^ using the recombinant human PD-L1/B7-H1 His-Tag protein (9049-B7-100, Bio-Techne) and HisPur Ni-NTA magnetic beads (Thermo Fisher Scientific).

The specificity of ^89^Zr-DFO-Atezolizumab binding was assessed in U87-MGvIII, GCGR-E55, BL6-NPE-IE, GL261, and GL261_PDL1KO_ cell lines. A detailed description of the procedure is given in the Supplementary Materials.

### ^89^Zr-DFO-Atezolizumab In Vivo Imaging and Ex Vivo Studies

PET/CT imaging studies were conducted using an Albira PET/SPECT/CT imaging system (Bruker). PET/CT scans were acquired 24, 48, and 72 hours post-intravenous injection of ^89^Zr-DFO-Atezolizumab (111 μg Atezolizumab, 1.72-1.96 MBq/mouse in 100 μL). The detailed imaging and data analysis protocols are given in Supplementary Materials. For biodistribution studies, blood, major organs, and tumors were collected, and weighed. The radioactive content was measured by γ-counting. The decay-corrected data were expressed as a percentage of injected dose per gram of tissue (%ID/g; *n* = 4 ± SD).

### Ex vivo Histology and Quantification

Formalin-fixed tumors (10%, v/v) were embedded in paraffin, sectioned into 5-µm-thick slices, and mounted on microscope slides. For H&E, sections were stained with Gill’s hematoxylin (Pioneer Research Chemicals). For IHC, the detailed staining procedures with the various antibodies are described in Supplementary Materials. Slides were digitally processed using the Nanozoomer-XR slide scanner (Hamamatsu Photonics). Image analysis and digital scoring of PD-L1 expression was performed using QuPath (Queen’s University).^23^

## Clinical Methods

### Patient Selection and Study Design

The imaging studies involved eight adult patients with newly diagnosed GBM based on the 2021 WHO Classification of Tumors of the Central Nervous.^24^ Inclusion and exclusion criteria are summarized in **Supplementary Table S2**. Written informed consent was obtained prior to the study enrollment from all participants and was conducted in accordance with the Declaration of Helsinki. The studies were approved by the local Institutional Review Board (Maria Sklodowska-Curie National Research Institute of Oncology) and were conducted concurrently with a companion single-center, open-label, randomized study evaluating the safety and efficacy of neoadjuvant and adjuvant Pembrolizumab additional to standard chemo-radiotherapy treatment (Department of Neurosurgery, Medical University of Silesia). The studies complied with relevant ethical regulations approved by the Institutional Review Board at the Medical University of Silesia in Katowice, Poland, and the trial was registered at ClinicalTrials.gov (NCT05235737). As a result of randomization, five patients presented herein received 2 doses (200 mg each) of neoadjuvant Pembrolizumab by i.v. infusion 2 weeks apart (*neo*Pembrolizumab group). Pre- and post-contrast MR images were acquired and i.v. administration of ^89^Zr-DFO-Atezolizumab was performed 7 days before surgery. Three patients did not receive neoadjuvant Pembrolizumab and underwent only i.v. administration of ^89^Zr-DFO-Atezolizumab and pre- and post-contrast MRI (control group). All patients had adjuvant treatment, which included radiotherapy plus chemotherapy using temozolomide. More details about the studies are in Supplementary Materials.

### ^89^Zr-DFO-Atezolizumab Clinical Production

DFO-Atezolizumab and ^89^Zr-DFO-Atezolizumab were prepared from commercially available Atezolizumab (Tecentriq, 1200 mg/20 mL, Roche) at the Maria Sklodowska-Curie National Research Institute of Oncology in Gliwice, Poland, according to good manufacturing practice guidelines and under metal-free conditions. The conjugation and radiolabeling procedures are described in Supplementary Materials.

### ^89^Zr-DFO-Atezolizumab PET/CT

Patients were injected with 10 mg of unlabeled Atezolizumab and 37 MBq ± 10% of ^89^Zr-DFO-Atezolizumab (1 mg) via peripheral venous catheter by an infusion pump 7 days prior to surgery. The dose was selected based on the previously published protocol.^15^ After radioconjugate administration, patients were kept under observation for at least 2 hours to identify any infusion-related reactions. PET/CT scans were acquired 48 and 72 hours post-administration using a Siemens Biograph mCT Flow 40-slice PET/CT camera (Siemens Healthineers). The detailed imaging and data analysis protocols are given in Supplementary Materials.

### MRI Scans

Images were acquired with an MR scanner Siemens Magnetom Prisma 3T using a 20 channel head-neck-spine coil. The detailed imaging protocols are given in Supplementary Materials.

### Evaluation of IHC

FFPE tissue blocks obtained from the patients were cut into 4 μm-thick sections and stained routinely with hematoxylin and eosin (H&E). Initially, the status of the isocitrate dehydrogenase 1 (IDH1) mutation was assessed. All tumor samples were confirmed to be IDH1wt, therefore underwent further analysis. Automated IHC staining for PD-L1, CD4, and CD8 was subsequently performed using the Bench-Mark ULTRA platform (Roche) and Dako Autostainer Link 48 (Agilent) according to manufacturer protocols. The PD-L1 level was expressed using the tumor proportion score (TPS) and combined positive score (CPS), where TPS is the percentage of PD-L1-positive tumor cells compared to all tumor cells, while CPS is the percentage of PD-L1-positive tumor and inflammatory cells (ie, lymphocytes and macrophages) compared to all tumor cells. The detailed staining procedures with the various antibodies and quantification methods are described in Supplementary Materials.

### Statistical Analysis

Statistical analysis to determine the significance between 2 groups was performed using unpaired T-tests, and significance was considered for  *p<* 0.05 (*), *p* < .01 (**), *p* < .001 (***), and *p* < .0001 (****). The linear relationship between PD-L1 IHC and radioconjugate uptake quantification was evaluated using the Pearson correlation coefficient. Error bars are representative of standard deviation. All statistical analyses were performed using GraphPad (Version 9).

## Results

### Targeting Properties of Atezolizumab-Based Conjugates

**Figure 1.**
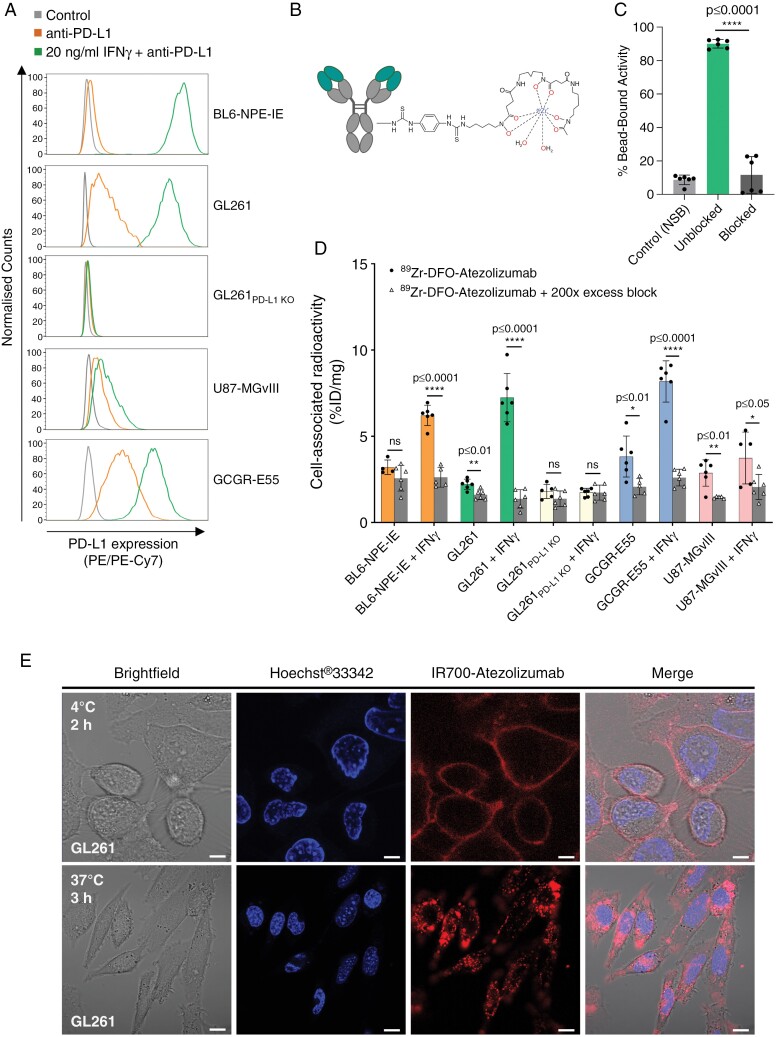
**(A)** Representative flow cytometry histograms showing PD-L1 expression in murine and human GBM cell lines with and without IFN-γ stimulation. **(B)** Schematic illustration of the ^89^Zr-DFO-Atezolizumab chemical structure. **(C)** Immunoreactive fraction of ^89^Zr-DFO-Atezolizumab in blocked and unblocked conditions (*n* = 6). **(D)** Binding specificity of ^89^Zr-DFO-Atezolizumab (1 nM) in murine and human GBM cell lines with and without IFN-γ stimulation (*n* = 6). **(E)** Representative confocal immunofluorescence images demonstrating IR700-Atezolizumab binding to PD-L1 in GL261 cells stimulated with IFN-γ at 4 °C and 37 °C; and nuclei visualization with Hoechst^®^33342 staining (4 °C, Scale bar: 5 μm, Magnification: × 63; 37 °C, Scale bar: 10 μm, Magnification: × 82).

Initially, we evaluated PD-L1 expression levels in selected human and murine GBM cell lines by flow cytometry. The analysis showed high levels of PD-L1 expression in GL261 and GCGR-E55 cells, and medium-to-low target expression in U87-MGvIII and BL6-NPE-IE, respectively. Minimal-to-no PD-L1 expression was confirmed in GL261_PD-L1KO_ cells. Incubation of cells with IFN-γ, a cytokine known to stimulate PD-L1 expression on tumor cells, substantially increased the ligand level in BL6-NPE-IE, GL261, and GCGR-E55, whilst no changes were observed in GL261_PD-L1KO_ cells (**Figure 1A**). The humanized IgG1 mAb targeting PD-L1, Atezolizumab, was radiolabeled with ^89^Zr (**Figure 1B**) and characterized in vitro. The ^89^Zr-DFO-Atezolizumab was produced with a radiochemical purity (RCP) >99%, a decay corrected radiochemical yield (RCY) of 57%–69% and a 0.18–0.20 MBq/µg apparent specific activity (26–29.6 MBq/nmol apparent molar activity) (**Supplementary Figure S1A and S1B**). When used in a rapid bead-based radioimmunoassay, the radioconjugate showed a target-binding fraction of 90% ± 2.5%, indicating good retention of the target recognition properties (**Figure 1C**). The ^89^Zr-DFO-Atezolizumab specificity binding studies showed a correlation between the cell-associated radioactivity and level of PD-L1 expression in human and murine GBM cells as seen by flow cytometry (**Figure 1D**). No difference was found in GL261_PDL1KO_ cells (negative control). Importantly, the radioactivity signal decreased to the baseline when cells were pre-treated with 200-fold molar excess of unlabeled Atezolizumab, confirming the ^89^Zr-DFO-Atezolizumab specificity for the target (BL6-NPE-IE: 6.2 vs 2.6 %ID/mg; GL261: 7.2 vs 1.4 %ID/mg; GCGR-E55: 8.2 vs 2.6 %ID/mg; U87-MGvIII: 3.7 vs 2.1 %ID/mg) (**Figure 1D**). To corroborate the specific targeting of Atezolizumab for PD-L1, IFN-γ-stimulated GL261 cells were incubated with IR700-Atezolizumab. Confocal microscopy detected a membrane-localized fluorescent signal of IR700-Atezolizumab after 2 hours of incubation at 4 °C in GL261, and a large amount of internalized conjugate after 3 hours of incubation at 37 °C (**Figure 1E**). There was no uptake of IR700-Atezolizumab in GL261_PD-L1KO_ cells after 2 hours of incubation at 37 °C (**Supplementary Figure S2A**). The specificity of binding studies performed with IR700-Atezolizumab demonstrated complementary median fluorescence intensity (MFI) of IR700-Atezolizumab with PD-L1 expression levels measured in a panel of human and murine GBM cells. In addition, pre-blocking with unlabeled Atezolizumab significantly decreased MFI to baseline (**Supplementary Figure S2B**).

### BBB Permeability Assessment

**Figure 2.**
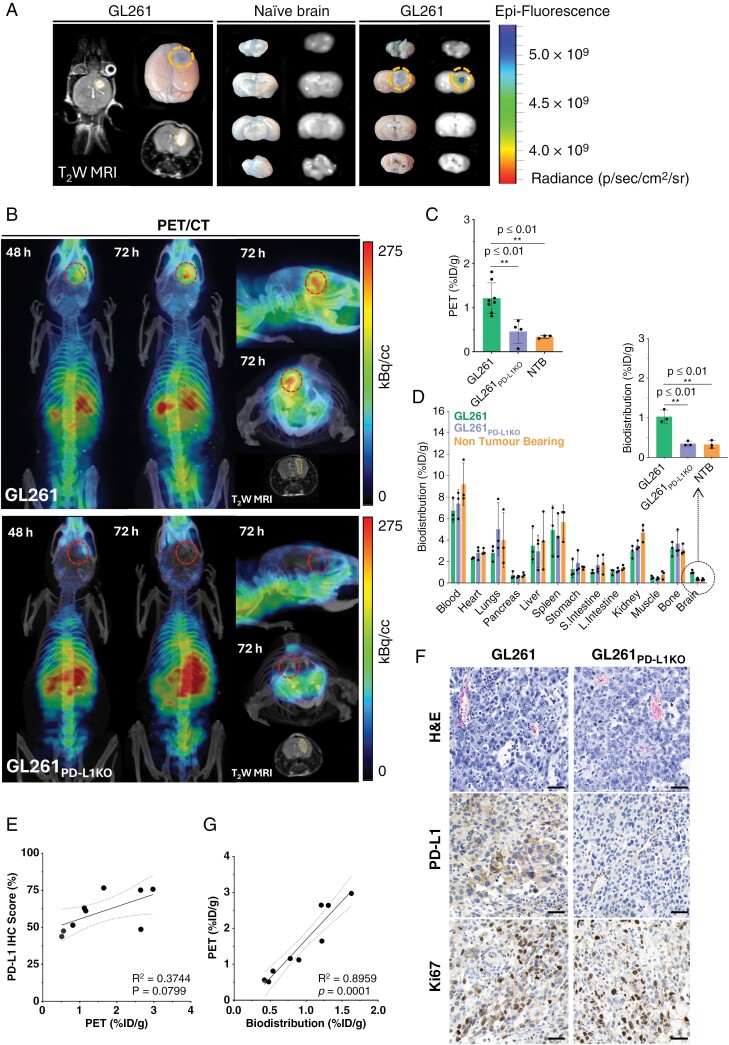
**(A)** Representative photographic and T_2_W MR images of GL261 tumor-bearing brains following i.v. injection with 1% Evans Blue (left panel). Photographic and IVIS fluorescent images of naïve and GL261 tumor-bearing brain slices (4 mm) following i.v. injection with 1% Evans Blue (middle and right panels). Evans Blue fluorescence was captured with 640 nm excitation and 680 nm emission filters. **(B)** Representative PET images of ^89^Zr-DFO-Atezolizumab uptake in mice-bearing orthotopic GL261 and GL261_PD-L1 KO_ at 48 and 72 hours post-injection. Anatomical visualization of tumors was performed by T_2_W MRI prior to ^89^Zr-DFO-Atezolizumab administration. **(C)** PET quantification (%ID/g) of ^89^Zr-DFO-Ateolizumab uptake in orthotopic GL261 (*n* = 8) and GL261_PD-L1 KO_ (*n* = 4) tumors, and naïve mouse brains (*n* = 3). **(D)** Ex vivo biodistribution of naïve mice and mice-bearing orthotopic GL261 and GL261_PD-L1 KO_ tumors 72 hours post-injection with ^89^Zr-DFO-Atezolizumab (*n* = 3). **(E)** Linear correlation between PET-quantified ^89^Zr-DFO-Atezolizumab uptake and PD-L1-positive IHC score (R_2_ = 0.3744). **(F)** Linear correlation between PET-quantified and biodistribution-quantified ^89^Zr-DFO-Atezolizumab uptake (R_2_ = 0.8959). **(G)** Representative images of H&E, PD-L1, and Ki67 IHC stained GL261 and GL261_PD-L1 KO_ tumors (Scale bar: 50 μm, Magnification: × 40).

The BBB integrity in both naïve and GL261 brain tumor-bearing mice was assessed using Evans Blue perfusion. Fluorescent imaging of isolated brains with GL261 tumors showed the presence of highly localized fluorescent areas. MRI scans of the same brains confirmed the co-localization of the tumors and the zones of Evans Blue extravasation (**Figure 2A**). No fluorescent regions were detected in naïve brains. These results suggested a certain degree of disruption of the BBB within the tumor region which could promote the brain tumor accessibility to molecules like mAbs.

### Preclinical In Vivo PET imaging With ^89^Zr-DFO-Atezolizumab

Owing to its human and mouse cross-reactivity, we investigated ^89^Zr-DFO-Atezolizumab in immunodeficient human GBM and immunocompetent murine GBM models for PD-L1 detection in tumors and healthy tissues to complement its utility in the clinical setting. ^89^Zr-DFO-Atezolizumab (111 µg of protein, 1.72–1.96 MBq/mouse) was intravenously injected in mice with MRI-confirmed U87-MGvIII, GL261 and GL261_PD-L1KO_ brain tumors. PET/CT images demonstrated focal tumor uptake as early as 24 hours p.i. and allowed for an accurate delineation of volumes of interest at 48 and 72 hours for GL261 and U87-MGvIII tumors, respectively (**Figure 2B** and **Supplementary Figure S3A**). A negligible signal was found in GL261_PD-L1KO_ tumors confirming the specificity of ^89^Zr-DFO-Atezolizumab to PD-L1 in vivo (**Figure 2B**). PET-based uptake quantification presented significantly higher uptake in U87-MGvIII and GL261 brain tumors compared to non-tumor-bearing (NTB) mice (U87-MGvIII vs NTB brains: 2.4 ± 0.8 %ID/g vs 0.5 ± 0.3 %ID/g, *p* *= *0.0205; GL261 vs NTB brains: 1.2 ± 0.3 %ID/g vs 0.3 ± 0.04 %ID/g,  *p= *0.0023) or mice-bearing PD-L1 negative tumors (GL261 vs GL261_PD-L1KO_: 1.2 ± 0.3 %ID/g vs 0.5 ± 0.3 %ID/g, *p* *= *0.0037) (**Figure 2C** and **Supplementary Figure S3B**). The ex vivo biodistribution of ^89^Zr-DFO-Atezolizumab revealed significantly higher uptake in U87-MGvIII and GL261 brain tumors compared to NTB mice (U87-MGvIII vs NTB brains: 1.4 ± 0.2 %ID/g vs 0.2 ± 0.1 %ID/g, *p* *= *0.0012; GL261 vs NTB brains: 1.0 ± 0.2 %ID/g vs 0.3 ± 0.1 %ID/g, *p = *0.0032) or mice-bearing PD-L1 negative tumors (GL261 vs GL261_PD-L1KO_: 1.0 ± 0.2 %ID/g vs 0.3 ± 0.1 %ID/g,  *p = *0.0026) (**Figure 2D and****Supplementary Figure S3C**). Amongst the other organs/tissues, a high uptake was observed in the spleen, an immune-cell-rich organ, as well as in the liver (U87-MGvIII: 2.4 ± 0.2 %ID/g, GL261: 3.8 ± 0.9 %ID/g) and kidneys, that are involved in the metabolism and clearance of the radioconjugate (U87-MGvIII: 3.5 ± 0.3 %ID/g, GL261: 3.8 ± 1.2 %ID/g). The blood signal was markedly higher in immunocompetent mice (7.8–11.5 %ID/g) compared to immunodeficient mice (4.1–5.5 %ID/g) presumably due to a larger population of circulating PD-L1-expressing peripheral blood mononuclear cells. Bone uptake in all mice ranged from 1.4%ID/g to 4.0 %ID/g, indicating some release of bone-seeking ^89^Zr4^+^ from the radioconjugate (**Figure 2D**). IHC scoring moderately correlated with ^89^Zr-DFO-Atezolizumab PET brain uptake quantification (R_2_ = 0.3744, *p* = 0.0799; **Figure 2E**). PET-based uptake quantification also demonstrated a linear correlation with ^89^Zr-DFO-Atezolizumab uptake ascertained from biodistribution studies (R_2_ = 0.8959, *p *= 0.0001; **Figure 2G**). Moreover, ex vivo H&E, Ki67 and PD-L1 IHC staining of brain sections confirmed tumor morphology, proliferation, and PD-L1 expression in all 3 tumor models (**Figure 2F** and **Supplementary Figure S3D**).

### Ex Vivo GL261 Immunophenotyping

**Figure 3.**
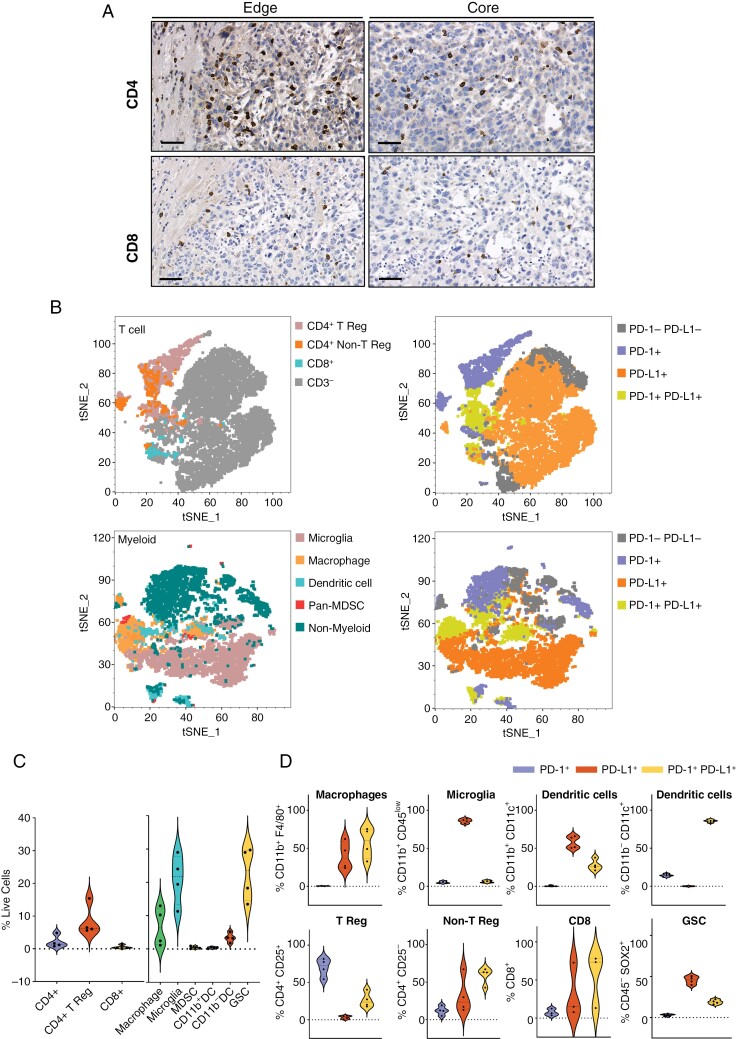
**(A)** Representative CD4 and CD8 IHC stained GL261 tumors (Scale bar: 50 μm, Magnification: × 40). **(B)** Composition of myeloid and T cell populations and PD-1/PD-L1 expression using t-SNE maps of CD45 + cells from GL261 tumors (*n* = 4). **(D)** Flow cytometry quantification of myeloid and T cell populations as a frequency of live cells from GL261 tumors (*n* = 4). **(E)** Flow cytometry quantification of PD-1/PD-L1 expression as a frequency of gated myeloid and T cell populations (*n* = 4).

T cell infiltration within GL261 tumors was examined histologically by ex vivo CD4 and CD8 IHC staining. Tumor edge and core regions revealed higher CD4-positively stained cells compared with CD8-positively stained cells, whilst a higher abundance of CD4-positively stained cells was present at the tumor edge regions compared to core regions (**Figure 3A**). Immunophenotyping of GL261 tumors was performed using flow cytometry to identify the composition of T cell and myeloid populations within the TME, and their respective levels of PD-1 and PD-L1 expression (**Supplementary Figures S4A** and **S4B****).** T-SNE cluster mapping indicated lymphoid populations were highly enriched with PD-1^+^ T reg cells, and myeloid cell populations were mostly comprised of PD-L1^+^ microglia and macrophages (**Figure 3B**). T reg cells were almost exclusively expressing PD-1 (PD-1^+^ vs PD-L1^+^ vs PD-1^+^ PD-L1^+^; 70.3% ± 12.1% vs 3.2% ± 2.2% vs 26.4% ± 10.1%). Myeloid microglia expressed higher levels of PD-L1 compared to PD-1 (PD-1^+^ vs PD-L1^+^ vs PD-1^+^ PD-L1^+^; 5.2% ± 1.4% vs 86.0% ± 2.8% vs 6.1% ± 1.5%) whereas macrophages (PD-1^+^ vs PD-L1^+^ vs PD-1^+^ PD-L1^+^; 0.3% ± 0.3% vs 40.1% ± 18.0% vs 57.4% ± 20.2%) populations were expressing both PD-1 and PD-L1. SOX2^+^ CD45^-^ cells (identified as glioma-stem cells) accounted for 22.6% ± 8.0% of all live cells and were largely PD-L1 positive cells (PD-1^+^ vs PD-L1^+^ vs PD-1^+^ PD-L1^+^; 3.1% ± 1.2% vs 45.6% ± 4.8% vs 19.0% ± 2.7%; **Figure 3C and D**).

### Immuno-PET and ^89^Zr-DFO-Atezolizumab Biodistribution

**Figure 4.**
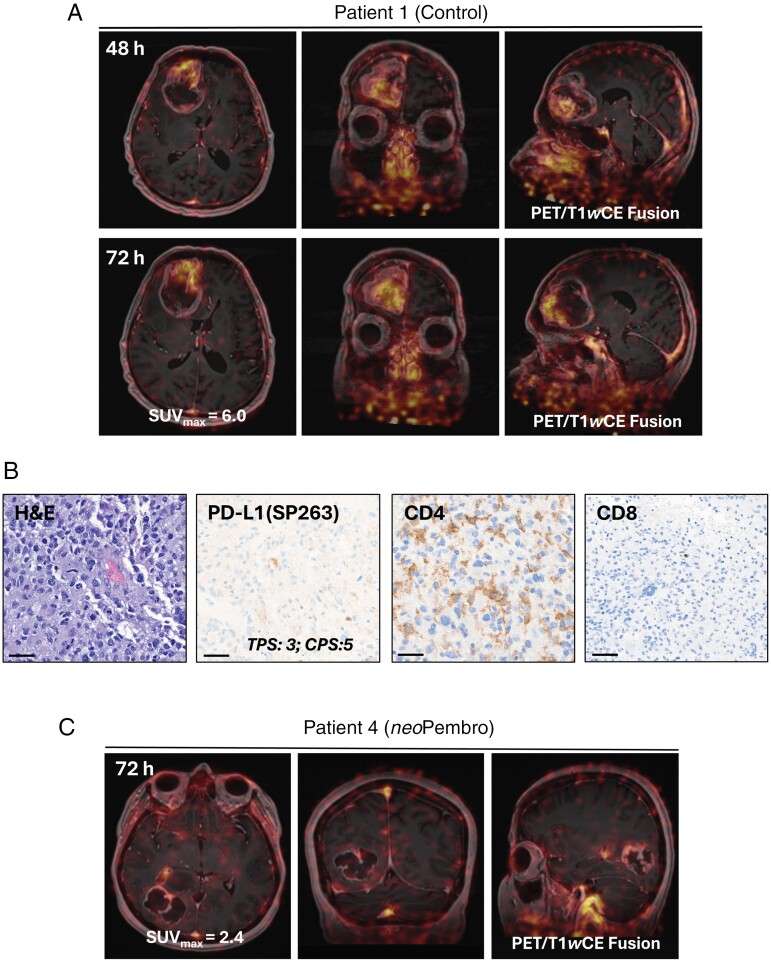

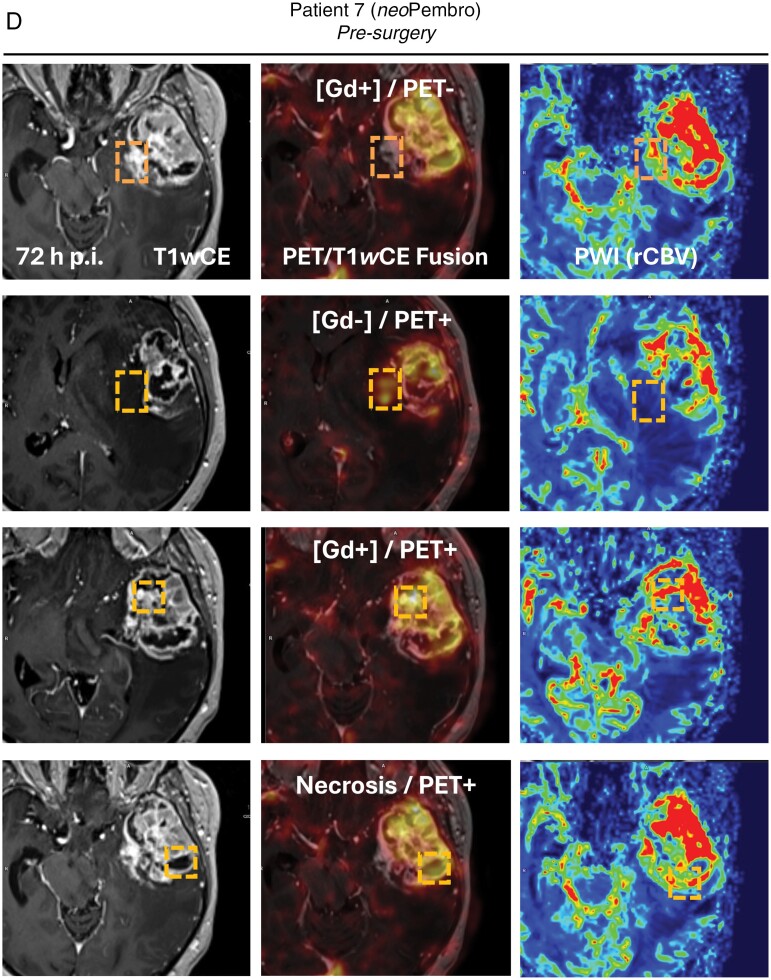
**(A)** Representative PET/contrast-enhanced T_1_W (T1*w*CE) MR fusion images taken pre-surgery from patient 1 (control) at 48 and 72 hours post-injection of ^89^Zr-DFO-Atezolizumab. **(B)** Representative H&E and CD4, CD8, and PD-L1 IHC staining from the patient 1 resected tumor specimen (Scale bar: 50 μm, Magnification: × 38). **(C)** Representative PET/T1*w*CE MR fusion images taken pre-surgery from patient 4 (*neo*Pembrolizumab) at 72 hours post-injection of ^89^Zr-DFO-Atezolizumab. **(D)** Representative T1*w*CE MR fusion image only (left column), fusion with PET (middle column), and perfusion-weighted imaging (PWI) (right column) from patient 7 demonstrating heterogeneous ^89^Zr-DFO-Atezolizumab uptake and vascular permeability. The dashed boxes highlight either gadolinium positive–negative and/or PET-positive–negative regions of interest across corresponding T1*w*CE MR, PET/T1*w*CE MR fusion, and PWI image slices.

**Figure 5.**
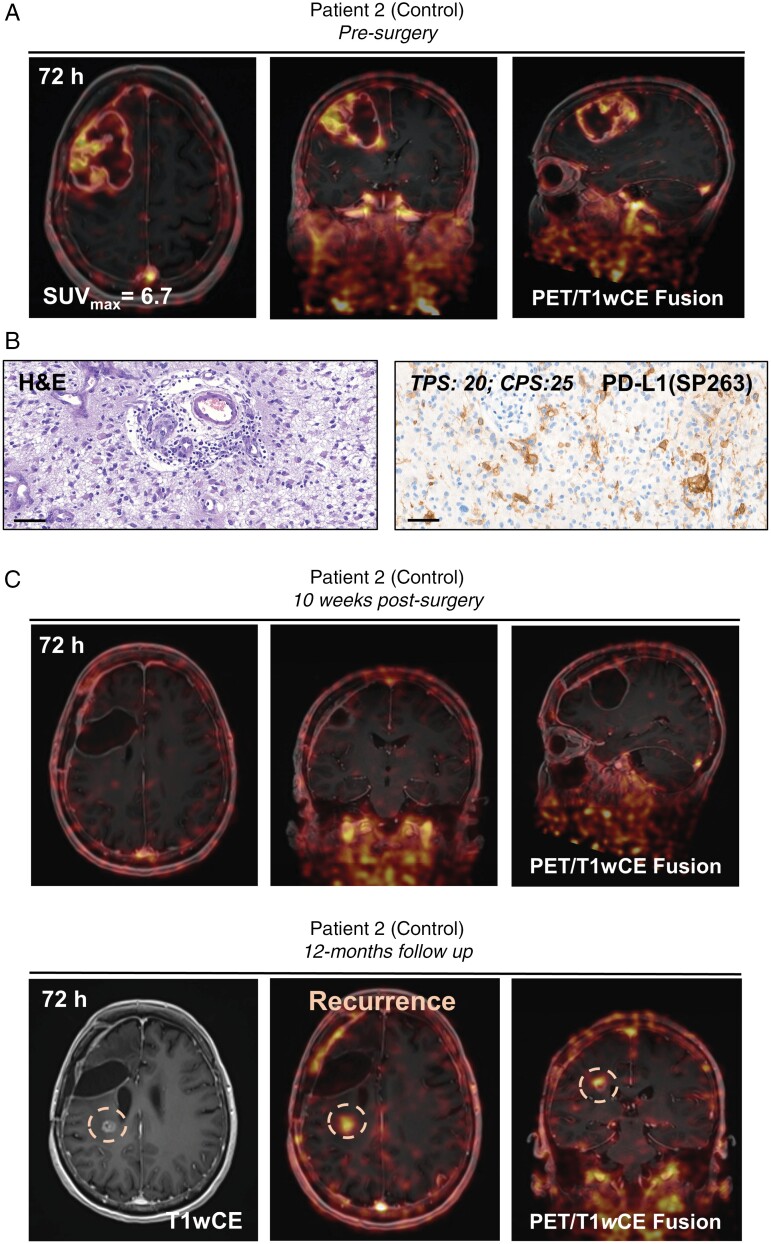
**(A)** Representative PET/contrast-enhanced T_1_W (T1*w*CE) MR fusion images taken pre-surgery from patient 2 (control) at 72 hours post-injection of ^89^Zr-DFO-Atezolizumab. **(B)** Representative H&E and PD-L1 IHC staining from the patient 1 resected tumor specimen (Scale bar: 50 μm, Magnification: × 38). **(C)** Representative PET/T1*w*CE MR fusion images taken at 10 weeks and 12 months post-surgery from patient 2 at 72 hours post-injection of ^89^Zr-DFO-Atezolizumab.

**Figure 6.**
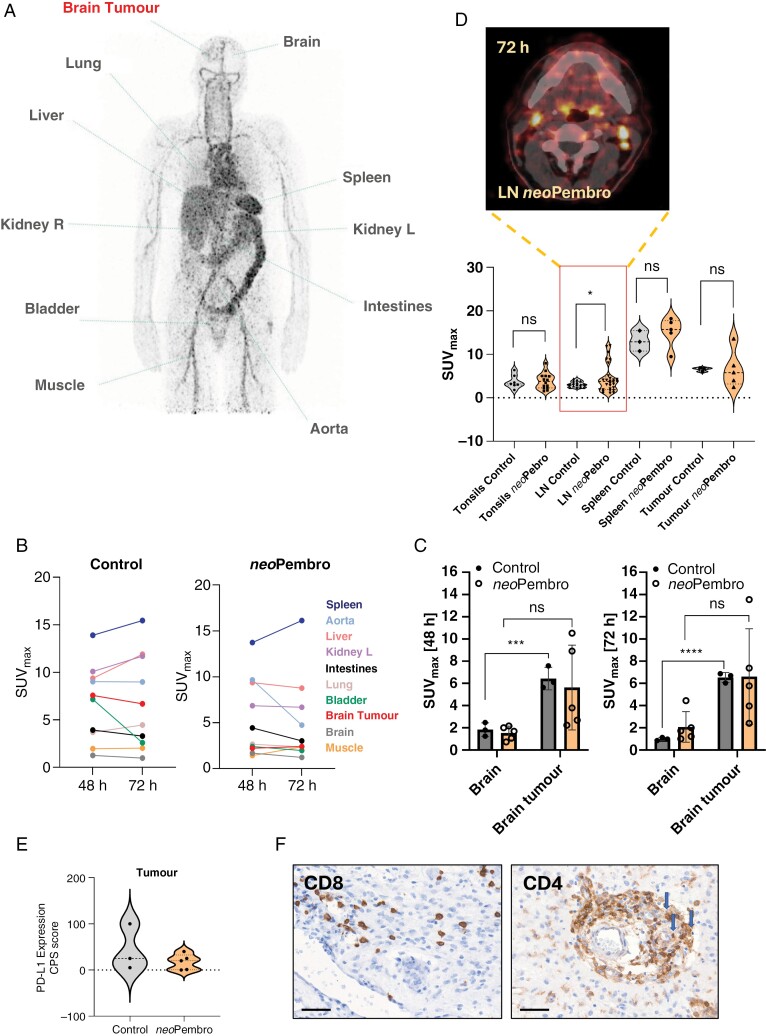
**(A)** Whole-body MIP of ^89^Zr-DFO-Atezolizumab uptake in patient 2 (control) at 72 hours post-injection. **(B)** Representative biodistribution of ^89^Zr-DFO-Atezolizumab uptake quantified using SUV_max_ from patient 2 (control). **(C)** Comparison of SUV_max_ between healthy brain and tumor at 48 and 72 hours post-injection of ^89^Zr-DFO-Atezolizumab in control (*n* = 3) and *neo*Pembrolizumab arms (*n* = 5). **(D)** Comparison of SUV_max_ taken from the tonsils, tumor-draining lymph nodes, spleen, and tumor (control/*neo*Pembrolizumab). Representative PET/CT image of ^89^Zr-DFO-Atezolizumab uptake in the tumor-draining lymph nodes in patient 5 treated with neoadjuvant Pembrolizumab at 72 hours post-injection. **(E)** Combined positive scoring (CPS) of PD-L1 expression from control (*n* = 3) and *neo*Pembrolizumab-treated patient tumor resection tissues (*n* = 5). **(F)** Representative CD4 and CD8 IHC staining from patient 2 in the control group (Scale bar: 50 μm, Magnification: × 38).

Eight patients with MRI-confirmed newly diagnosed GBM were enrolled in the study (2 female and 6 male between 42 and 70 years old); five patients received neoadjuvant Pembrolizumab, and the treatment was well tolerated. All patients received ^89^Zr-DFO-Atezolizumab (~1 mg protein dose) co-injected with 10 mg of unlabeled Atezolizumab. The dose was selected based on the previously reported study.^15^ The mean radioactivity at the time of injection was 36.63 ± 1.25 MBq. No infusion-related reactions or adverse events were observed post-radiotracer administration. In patients from the control group (*n* = 3), PET scans demonstrated heterogeneous but prominent radiotracer uptake in the avid part of the tumor as well as in regions surrounding the tumor rim which presented both gadolinium (Gd) enhancement and absence of the contrast agent on T_1_W MRI (**Figures 4A and 5A**, **Supplementary Figure S5A**). The whole-body PET image showing ^89^Zr-DFO-Atezolizumab distribution is presented in **Figure 6A**. The tumor uptake of ^89^Zr-DFO-Atezolizumab administration quantified from the whole-body scans resulted in the maximum standardized uptake value (SUV_max_) of 6.6 ± 0.85 and 5.3 ± 2.5 at 48 and 72 hours, respectively, and was significantly higher from the uptake measured in the healthy part of the brain (SUV_max_: 1.6 ± 0.3 at 48 hours and 1.0 ± 0.1 at 72 hours; **Figure 6C**). In one patient, we found a high radiotracer accumulation in the sinuses due to clinically observed inflammation and most likely binding of ^89^Zr-DFO-Atezolizumab to PD-L1-expressing immune cells present in that region (**Figure 4A**). Moreover, there was no significant uptake of the radiotracer in the brain on the follow-up scans acquired post-chemoradiotherapy (**Figure 5C and****Supplementary Figure S6B**). Interestingly, the tumor uptake was variable in patients who received neoadjuvant treatment. In three patients, the uptake was lower (SUV_max_: 2.9 ± 0.8 at 48 hours p.i. and 4.0 ± 0.8 at 72 hours p.i.) compared to patients in the control group. However, in two patients, uptake was relatively higher (SUV_max_: 9 and 10.5 at 48 hours p.i. and 13.5 and 7.5 at 72 hours p.i.). The quantitative analyses of the radioconjugate uptake are summarized in **Figure 6C**. There were no significant differences in the radioconjugate uptake measured in individual organs over time. The SUV_max_ in the circulation (measured in the abdominal aorta) was 6.9 ± 1.8 at 48 hours and 6.3 ± 2.3 at 72 hours p.i. in the control group (**Supplementary Figures S7A** and **S7B**). Furthermore, the organ distribution showed a high SUV_max_ in the liver and kidneys that reflected the metabolism and excretion of the radiotracer. As expected, all patients showed high spleen uptake with a mean SUV_max_ at 72 hours p.i. of 17.0 ± 2.3 in the control group and 17.7 ± 3.4 in the neoadjuvant group due to radioconjugate binding to PD-L1 positive immune cells (**Supplementary Figures S7A** and **S7B**). Tumor-draining lymph nodes were better visualized in patients who received neoadjuvant treatment which could indicate the activation of the body’s immune system in response to PD-1 inhibitor, despite significant variability between patients (**Figure 6D**). Low ^89^Zr-DFO-Atezolizumab uptake was observed in lung, muscle, and healthy part of the brain tissue. Up until the manuscript submission, six patients showed stable disease, one patient had progressed, and one patient died (because of TMZ complications). The median follow-up was 12.5 months in the whole patient population. In the patient whose disease has progressed, ^89^Zr-DFO-Atezolizumab PET showed focal radiotracer accumulation correlated with high signal intensity from a new lesion in the right frontal lobe seen at MRI, located outside of the tumor resection cavity (**Figure 5C**).

We observed major heterogeneity in PD-L1 IHC staining (**Figure 6E**). No correlation between CPS score and SUV_max_ was found. We also performed CD8 and CD4 staining of tumor samples collected from the PET “hot” regions where perivascular and intratumoral presence of CD8 and CD4 T cells was observed (**Figure 6F**). In the majority of cases, PD-L1-positive patterns were associated with moderate lymphocyte intensities. The quantitative analysis of PD-L1, CD4, and CD8 is summarized in **Supplementary Table S7**.

## Discussion

The development of immuno-PET radioconjugates to visualize tumor and whole-body PD-L1 expression, both prior to and during therapy, holds utmost significance in guiding patient management. This methodology has been successfully employed in clinical studies to evaluate the immune landscape of various tumor types.^25^ Nevertheless, in the context of GBM, immuno-PET has primarily been investigated in a preclinical setting until now. This study reports for the first time, to the best of our knowledge, the benefits of utilizing ^89^Zr-DFO-Atezolizumab to non-invasively map PD-L1 expression in newly diagnosed GBM tumors and immune-cell-rich organs over time, and in response to neoadjuvant immunotherapy.

Currently, it is well recognized that PD-L1 present in tumor cells, dendritic cells, and macrophages within the TME has a key role in mediating T cell immunosuppression and predicting the therapeutic outcome of PD-L1/PD-1 blockade.^26,27^ While high PD-L1 expression has been strongly correlated with an unfavorable prognosis for GBM patients, its effectiveness as a predictive marker for patient response to ICPIs has been largely unsuccessful. Interestingly, despite relatively high PD-L1 expression rates, which range from 6.1% to 88%.^6^ PD-L1/PD-1 inhibitors in GBM patients have yet to demonstrate a clear clinical benefit.^28,29^ The immunologically “cold” GBM phenotype likely limits their efficacy, suggesting that rational combinatorial therapies targeting immune suppression or enhancing BBB permeability may help convert “cold” tumors into “hot” ones, thereby improving the response to these drugs. This underscores the urgent need to introduce reliable methods for accurate PD-L1 evaluation. Currently, there is a lack of real-time monitoring methods for PD-L1 changes. IHC staining of PD-L1 has significant limitations, including the absence of consensus on antibody clones, criteria for PD-L1 positivity, and testing platforms. Additionally, studies often vary in design, with differences in retrospective versus prospective approaches, diverse patient cohorts, prior treatments, grading criteria, and comparisons between primary and highly resistant recurrent.^30–32^ For example, Heynckes et al. reported significant reductions in PD-L1 gene and protein expression in recurrent GBM compared to de novo GBM.^33^ Similarly, Berghoff et al. found that membranous PD-L1 protein expression is more frequent in newly diagnosed GBM than in matched recurrent GBM specimens.^34^ These findings, combined with the spatial and temporal heterogeneity of PD-L1 expression, which is dynamic and may be influenced by the distinct molecular characteristics of GBM subtypes and the chosen therapeutic regimens, further underscore the complexity of PD-L1 evaluation in GBM. For this reason, we explored the use of immuno-PET for whole tumor and body imaging as a complementary tool for PD-L1 assessment. We radiolabeled Atezolizumab with Zr-89 (t½ = 78.4 hours) due to its biological compatibility with the half-life of mAbs and relatively high image contrast provided by clearance from non-target expressing tissue.

The preclinical evaluation of the radioconjugate confirmed its high purity and stability. In addition, our in vitro studies using a panel of GBM cell lines demonstrated that ^89^Zr-DFO-Atezolizumab specifically targets human and murine PD-L1 epitopes in an expression-dependent manner. Clear accumulation of the radioconjugate in vivo was seen in mice-bearing human U87-MGvIII and murine GL261 tumors. Although PD-L1 expression was comparable between these 2 cell lines in vitro, uptake was moderately higher in the U87-MGvIII tumors, possibly due to differences between the use of mouse models. The lower blood uptake observed in immunocompromised mice with U87-MGvIII tumors, compared to immunocompetent mice with GL261 tumors, may be attributed to the absence of PD-L1-expressing peripheral mononuclear cells, leading to more ^89^Zr-DFO-Atezolizumab being available for binding to tumor epitopes. Furthermore, ex vivo immunophenotyping of these tumors presented a high abundance of PD-L1-expressing TAMs and GSCs, which relates to TAM infiltration seen in human mesenchymal GBM.^35^ Conversely, there was only negligible uptake in the GL261_PD-L1KO_ tumors, which appears to reflect endothelial cell PD-L1 expression as seen with IHC.

In our clinical studies, ^89^Zr-DFO-Atezolizumab was found to be safe and well tolerated with no infusion-related adverse events. The imaging results showed a high specificity of the radioconjugate to detect PD-L1-positive cells present within the tumor mass and in the whole body. The highest ^89^Zr-DFO-Atezolizumab SUV_max_ values were visualized in the control group compared to the individuals who received neoadjuvant Pembrolizumab treatment, although these differences were not statistically significant. Importantly, within the neoadjuvant group, we observed a markedly increased uptake in lymphoid tissues expressing PD-L1 (eg, spleen, lymph nodes). Even small lymph nodes (measuring 3 mm in the largest diameter) had enhanced ^89^Zr-DFO-Atezolizumab accumulation, suggesting that the imaging probe has a high sensitivity for PD-L1 on APCs. Interestingly, Bensch et al. also found increased uptake of ^89^Zr-Atezolizumab in the spleen and lymph nodes in response to PD-L1 blockade in bladder cancer, indicating systemic drug-induced changes in non-tumor sites caused by PD-1/PD-L1 blockade and highlighting potential utility for guiding future immunotherapy combination trials.^15^

Despite the encouraging results, several limitations in this study should be considered, many of which are inherent to the vast heterogeneity of GBM, its complex immunosuppressive mechanisms, and the use of immuno-PET imaging. Firstly, this proof-of-concept study involved a small participant cohort, which limited our ability to draw definitive conclusions from some of the available data, particularly regarding the lower expression levels of PD-L1 within the tumor post-neoadjuvant treatment. We anticipate that since PD-L1 upregulation can be driven by the influx of tumor antigen-specific PD-1^+^ TILs into the TME, blocking PD-1 on these cells might result in downregulation of PD-L1 expression due to fewer receptors being available for binding. Therefore, the future success of PD-1/PD-L1 inhibitors in GBM undoubtedly relies on further larger-scale prospective studies that more comprehensively evaluate the expression of PD-L1 and other biomarkers within the TME. Perhaps targeting TAMs and other myeloid-derived cells expressing potential biomarkers or molecules aiming to increase cytotoxic T cell infiltration should be explored in combination with PD-1/PD-L1.

Secondly, the relatively large molecular size of Atezolizumab results in slow uptake and elimination of the radioconjugate. This, in turn, makes routine clinical planning logistically challenging. Alternatively, smaller molecules like PD-L1 specific affibody molecule (Z_PD-L1_) could be considered, as they would allow for same-day imaging, providing greater convenience in a clinical setting.^13^ Another limitation identified in this study is the use of SUV for quantifying radioconjugate uptake. While SUV is widely used, it does not account for differences in plasma kinetics between patients. A recent study by Wijngaarden et al. proposed an alternative method using Patlak linearization (VT and Ki values), which more accurately quantifies irreversible uptake. Although the application of Patlak quantification to ^89^Zr immuno-PET agents in the brain requires further investigation, it could potentially offer a more precise accurate method of quantification in future studies.^36^

Finally, ^89^Zr-labeled antibodies are associated with an effective dose in the range of 20-40 mSv, which is relatively high and typically justifiable only for cancer patients. For non-oncological patients, the dose limit in Europe is 10 mSv.^37^ However, this high radiation exposure could be mitigated by using whole-body PET scanners, which have 40 times higher sensitivity compared to existing PET/CT systems.^38^ Moreover, considering the dismal 5-year survival rate of less than 5% in GBM patients, radiation exposure becomes a comparatively minor concern. The potential advantages offered by immuno-PET scans are likely to overwhelmingly outweigh the associated radiation risks.

## Conclusions

Our studies in both GBM mouse models and adult patients with newly diagnosed GBM patients may demonstrate the application of ^89^Zr-DFO-Atezolizumab PET imaging for spatial visualization of PD-L1 expression. Clinical studies are currently ongoing and with a larger cohort of patients we will be able to interrogate the role of ^89^Zr-DFO-Atezolizumab in detecting PD-L1 changes in response to neoadjuvant Pembrolizumab.

## Supplementary material

Supplementary material is available online at *Neuro-Oncology* (https://academic.oup.com/neuro-oncology).

noae190_suppl_Supplementary_Materials

## Data Availability

All original data from this manuscript will be made available upon reasonable request.

## References

[1] Wen PY , Reardon DA. Neuro-oncology in 2015: Progress in glioma diagnosis, classification and treatment. Nat Rev Neurol. 2016;12(2):69–70. 26782337 10.1038/nrneurol.2015.242

[2] Waldman AD , Fritz JM, Lenardo MJ. A guide to cancer immunotherapy: From T cell basic science to clinical practice. Nat Rev Immunol. 2020;20(11):651–668. 32433532 10.1038/s41577-020-0306-5 PMC7238960

[3] Simonds EF , Lu ED, Badillo O, et al Deep immune profiling reveals targetable mechanisms of immune evasion in immune checkpoint inhibitor-refractory glioblastoma. J ImmunoTher Cancer. 2021;9(6):e002181. 34083417 10.1136/jitc-2020-002181 PMC8183210

[4] Nduom EK , Wei J, Yaghi NK, et al PD-L1 expression and prognostic impact in glioblastoma. Neuro Oncol. 2016;18(2):195–205. 26323609 10.1093/neuonc/nov172 PMC4724183

[5] Berghoff AS , Kiesel B, Widhalm G, et al Programmed death ligand 1 expression and tumor-infiltrating lymphocytes in glioblastoma. Neuro Oncol. 2014;17(8):1064–1075. 25355681 10.1093/neuonc/nou307 PMC4490866

[6] Vimalathas G , Kristensen BW. Expression, prognostic significance and therapeutic implications of PD-L1 in gliomas. Neuropathol Appl Neurobiol. 2022;48(1):e12767. 34533233 10.1111/nan.12767 PMC9298327

[7] Wang Z , Zhang C, Liu X, et al Molecular and clinical characterization of PD-L1 expression at transcriptional level via 976 samples of brain glioma. Oncoimmunol. 2016;5(11):e1196310. 10.1080/2162402X.2016.1196310 PMC5139638 27999734

[8] Henrik Heiland D , Haaker G, Delev D, et al Comprehensive analysis of PD-L1 expression in glioblastoma multiforme. Oncotarget. 2017;8(26):42214–42225. 28178682 10.18632/oncotarget.15031 PMC5522061

[9] Reardon DA , Brandes AA, Omuro A, et al Effect of Nivolumab vs Bevacizumab in patients with recurrent glioblastoma: The Checkmate 143 phase 3 Randomized Clinical Trial. JAMA Oncol. 2020;6(7):1003–1010. 32437507 10.1001/jamaoncol.2020.1024 PMC7243167

[10] Omuro A , Brandes AA, Carpentier AF, et al Radiotherapy combined with nivolumab or temozolomide for newly diagnosed glioblastoma with unmethylated MGMT promoter: An international randomized phase III trial. Neuro Oncol. 2023;25(1):123–134. 35419607 10.1093/neuonc/noac099 PMC9825306

[11] Omuro A , Reardon DA, Sampson JH, et al Nivolumab plus radiotherapy with or without temozolomide in newly diagnosed glioblastoma: Results from exploratory phase I cohorts of CheckMate 143. Neurooncol. Adv.. 2022;4(1):vdac025. 35402913 10.1093/noajnl/vdac025 PMC8989388

[12] Cloughesy TF , Mochizuki AY, Orpilla JR, et al Neoadjuvant anti-PD-1 immunotherapy promotes a survival benefit with intratumoral and systemic immune responses in recurrent glioblastoma. Nat Med. 2019;25(3):477–486. 30742122 10.1038/s41591-018-0337-7 PMC6408961

[13] Sharma G , Braga MC, Da Pieve C, et al Immuno-PET imaging of tumour PD-L1 expression in glioblastoma. Cancers (Basel). 2023;15(12):3131. 37370741 10.3390/cancers15123131 PMC10295898

[14] Parakh S , Lee ST, Gan HK, Scott AM. Radiolabeled antibodies for cancer imaging and therapy. Cancers (Basel). 2022;14(6):1454. 35326605 10.3390/cancers14061454 PMC8946248

[15] Bensch F , van der Veen EL, Lub-de Hooge MN, et al Zr-89-atezolizumab imaging as a non-invasive approach to assess clinical response to PD-L1 blockade in cancer. Nat Med. 2018;24(12):1852–1858. 30478423 10.1038/s41591-018-0255-8

[16] Smit J , Borm FJ, Niemeijer AN, et al PD-L1 PET/CT imaging with radiolabeled durvalumab in patients with advanced-stage non-small cell lung cancer. J Nucl Med. 2022;63(5):686–693. 34385342 10.2967/jnumed.121.262473

[17] Kok IC , Hooiveld JS, van de Donk PP, et al Zr-89-pembrolizumab imaging as a non-invasive approach to assess clinical response to PD-1 blockade in cancer. Ann Oncol. 2022;33(1):80–88. 34736925 10.1016/j.annonc.2021.10.213

[18] Lee AH , Sun L, Mochizuki AY, et al Neoadjuvant PD-1 blockade induces T cell and cDC1 activation but fails to overcome the immunosuppressive tumor associated macrophages in recurrent glioblastoma. Nat Commun. 2021;12(1):6938. 34836966 10.1038/s41467-021-26940-2 PMC8626557

[19] Pollard SM , Yoshikawa K, Clarke ID, et al Glioma stem cell lines expanded in adherent culture have tumor-specific phenotypes and are suitable for chemical and genetic screens. Cell Stem Cell. 2009;4(6):568–580. 19497285 10.1016/j.stem.2009.03.014

[20] Workman P , Aboagye EO, Balkwill F, et al; Committee of the National Cancer Research Institute. Guidelines for the welfare and use of animals in cancer research. Br J Cancer. 2010;102(11):1555–1577. 20502460 10.1038/sj.bjc.6605642 PMC2883160

[21] Percie du Sert N , Hurst V, Ahluwalia A, et al The ARRIVE guidelines 2.0: Updated guidelines for reporting animal research. BMJ Open Sci. 2020;4(1):e100115. 10.1136/bmjos-2020-100115 PMC7610906 34095516

[22] Sharma SK , Lyashchenko SK, Park HA, et al A rapid bead-based radioligand binding assay for the determination of target-binding fraction and quality control of radiopharmaceuticals. Nucl Med Biol. 2019;71:32–38. 31128476 10.1016/j.nucmedbio.2019.04.005 PMC6599726

[23] Bankhead P , Loughrey MB, Fernández JA, et al QuPath: Open source software for digital pathology image analysis. Sci Rep-Uk. 2017;7(1):16878. 10.1038/s41598-017-17204-5 PMC5715110 29203879

[24] Berger TR , Wen PY, Lang-Orsini M, Chukwueke UN. World Health Organization 2021 classification of central nervous system tumors and implications for therapy for adult-type gliomas: A review. JAMA Oncol. 2022;8(10):1493–1501. 36006639 10.1001/jamaoncol.2022.2844

[25] Manafi-Farid R , Ataeinia B, Ranjbar S, et al ImmunoPET: Antibody-Based PET imaging in solid tumors. Front Med-Lausanne. 2022;9:916693. 35836956 10.3389/fmed.2022.916693 PMC9273828

[26] Peng Q , Qiu X, Zhang Z, et al PD-L1 on dendritic cells attenuates T cell activation and regulates response to immune checkpoint blockade. Nat Commun. 2020;11(1):4835. 32973173 10.1038/s41467-020-18570-x PMC7518441

[27] Liu YT , Zugazagoitia J, Ahmed FS, et al Immune cell PD-L1 colocalizes with macrophages and is associated with outcome in PD-1 pathway Blockade Therapy. Clin Cancer Res. 2020;26(4):970–977. 31615933 10.1158/1078-0432.CCR-19-1040 PMC7024671

[28] Khan F , Pang L, Dunterman M, et al Macrophages and microglia in glioblastoma: Heterogeneity, plasticity, and therapy. J Clin Invest. 2023;133(1):e163446. 36594466 10.1172/JCI163446 PMC9797335

[29] Chauhan P , Lokensgard JR. Glial cell expression of PD-L1. Int J Mol Sci . 2019;20(7):1677. 30987269 10.3390/ijms20071677 PMC6479336

[30] Akhtar M , Rashid S, Al-Bozom IA. PD-L1 immunostaining: What pathologists need to know. Diagn Pathol. 2021;16(1):94. 34689789 10.1186/s13000-021-01151-x PMC8543866

[31] Vranic S , Gatalica Z. PD-L1 testing by immunohistochemistry in immuno-oncology. Biomol Biomed. 2023;23(1):15–25. 35964287 10.17305/bjbms.2022.7953 PMC9901897

[32] Ma W , Gilligan BM, Yuan J, Li T. Current status and perspectives in translational biomarker research for PD-1/PD-L1 immune checkpoint blockade therapy. J Hematol Oncol. 2016;9(1):47. 27234522 10.1186/s13045-016-0277-y PMC4884396

[33] Heynckes S , Gaebelein A, Haaker G, et al Expression differences of programmed death ligand 1 in de-novo and recurrent glioblastoma multiforme. Oncotarget. 2017;8(43):74170–74177. 29088776 10.18632/oncotarget.18819 PMC5650331

[34] Berghoff AS , Kiesel B, Widhalm G, et al Programmed death ligand 1 expression and tumor-infiltrating lymphocytes in glioblastoma. Neuro Oncol. 2015;17(8):1064–1075. 25355681 10.1093/neuonc/nou307 PMC4490866

[35] Khan F , Pang LZ, Dunterman M, et al Macrophages and microglia in glioblastoma: Heterogeneity, plasticity, and therapy. J Clin Investig. 2023;133(1):e163446. 36594466 10.1172/JCI163446 PMC9797335

[36] Wijngaarden JE , Huisman MC, Jauw YWS, et al Validation of simplified uptake measures against dynamic Patlak K for quantification of lesional Zr-Immuno-PET antibody uptake. Eur J Nucl Med Mol I. 2023;50(7):1897–1905. 10.1007/s00259-023-06151-1 PMC10199860 36820891

[37] van Dongen G , Beaino W, Windhorst AD, et al The Role of (89)Zr-Immuno-PET in navigating and derisking the development of biopharmaceuticals. J Nucl Med. 2021;62(4):438–445. 33277395 10.2967/jnumed.119.239558

[38] Cherry SR , Jones T, Karp JS, et al Total-body PET: maximizing sensitivity to create new opportunities for clinical research and patient care. J Nucl Med. 2018;59(1):3–12. 28935835 10.2967/jnumed.116.184028 PMC5750522

