# Supplementary material for: Imaging PD-L1 in the brain—Journey from the lab to the clinic
Source: Neuro Oncol. 2024 Oct 28;27(2):567–82. doi: 10.1093/neuonc/noae190 (PMC11812043; doi:10.1093/neuonc/noae190)
Supplement: noae190_suppl_Supplementary_Materials [file noae190_suppl_supplementary_materials.docx]

**Supplementary Materials**

**Title: Imaging PD-L1 in the brain – journey from the lab to the clinic**

**Authors:** Dawoud Dar^1^, Magdalena Rodak^2^, Chiara Da Pieve^1^, Izabela Gorczewska^3^, **Gitanjali Sharma**^1^, Ewa Chmielik^4^, Marcin Niedbala^5^, Pawel Bzowski^4^, Andrea d’Amico^4^, Barbara Bobek-Bilewicz^6^ Elzbieta Nowicka^7^, Rafal Tarnawski^7^, Wojciech Kaspera^5*^ & Gabriela Kramer-Marek^1,2*^

*^1^Institute of Cancer Research, Department of Radiotherapy and Imaging, London, United Kingdom*

*^2^Maria Sklodowska-Curie National Research Institute of Oncology, Department of Radiopharmacy and Preclinical PET Imaging, Gliwice, Poland*

*^3^Maria Sklodowska-Curie National Research Institute of Oncology, Department of Nuclear Medicine and Endocrine Oncology, Gliwice, Poland*

*^4^Maria Sklodowska-Curie National Research Institute of Oncology, Department of Tumour Pathology, Gliwice, Poland*

*^5^Medical University of Silesia, Department of Neurosurgery, Katowice, Poland*

*^6^Maria Sklodowska-Curie National Research Institute of Oncology, Department of Radiology and Imaging Diagnostic, Gliwice, Poland*

*^7^Maria Sklodowska-Curie National Research Institute of Oncology, Department of IIIrd Radiotherapy and Chemotherapy, Gliwice, Poland*

**Contents:**

**Materials and Methods**

**Figure S1** HPLC chromatograms of Atezolizumab, DFO-Atezolizuamb and ^89^Zr-DFO-Atezolizumab

**Figure S2** Confocal immunofluorescence and flow cytometry analysis of IR700-Atezolizumab Specificity.

**Figure S3** PET scans of orthotopic U87-MGvIII tumours, and ex vivo biodistribution and histopathology.

**Figure S4** Immunophenotyping gating strategy for T cell and myeloid populations.

**Figure S5** PET scans and histopathology of patient 3 in the control arm

**Figure S6** PET scans and histopathology of patient 5 in the neoadjuvant Pembrolizumab arm

**Figure S7 SUV_max_ biodistribution of ^89^Zr-DFO-Atezolizumab uptake in patients in the control and neoadjuvant Pembrolizumab arm**

**Table 1** Primary antibodies used for preclinical IHC staining

**Table 2** Inclusion and exclusion criteria for patients enrolled in the trial

**Table 3** Release specifications of ^89^Zr-DFO-Atezolizumab

**Table 4** CT acquisition parameters for patient scans

**Table 5** PET acquisition parameters for patient scans

**Table 6** Antibodies used for staining patient samples

**References**

**Materials and Methods:**

**Cell Culture**

The human GCGR-E55 and the murine BL6-NPE-IE GBM cell lines were cultured in DMEM/Nutrient Mixture F-12/Ham supplemented with glucose, MEM NEAA, BSA, 2-mercaptoethanol, N2, B27 (Thermo Fisher Scientific, Loughborough, UK), mouse EGF, human FGF (10 ng/ml, PeproTech, Cranbury, USA) and laminin (2 mg/ml, Cultrex® 3D Culture Matrix Laminin I, Trevigen, Gaithersburg, USA)^1^.

**Mouse Models**

Female athymic nude mice (crl:NU(NCR)-Foxn1^nu^) and female C57BL/6J (6-8 weeks old) were obtained from Charles River Laboratories (Harlow, UK). Animals were housed in individually ventilated cages (Nexgen, Allentown, USA) with an area of 500 cm^2^ and a maximum of 5 animals per cage, temperature of 21±1°C, humidity of 55±10%, 12:12 h of dark:light cycles, and given food (Labdiet, St. Louis, US) and water *ad libitum.* Environmental enrichment included bedding substrate of Corn Cob 6/8, Aspen chew stick 5×1×1 cm^3^ (Datesand, Stockport, UK) and one Bed r’nest paper wool nesting material (Datesand, Stockport, UK).

For orthotopic cell implantation, animals were anaesthetised with gaseous isoflurane (3-4% induction and 1-2% maintenance v/v in O_2_ at 1 L/min, ISOFLURIN^®^, Barcelona, Spain). An incision was made to expose the skull and H_2_0_2_ (1% v/v) was applied to clear connective tissue and visualise the bregma. The injection site was stationed +2 mm lateral and -1 mm anterior to the bregma where the skull was drilled. U87-MGvIII, GL261, or GL261_PDL1KO_ cells were stereotactically injected 3.2 mm deep from the surface of the skull at 1 μL/min. Tumour growth was monitored with the 1T M3™ MRI system (Aspect Imaging, Shoham, Israel) using a T_2_W sequence (1 mm slice thickness, TR 4000 ms, TE 62.47 ms).

**Preparation of IR700-Atezolizumab**

Atezolizumab solution (Tecentriq^®^, 1200 mg/20 mL, Roche, San Francisco, US) was buffer exchanged in 0.1 M phosphate buffer pH 8.6 using a Pierce protein concentrator (50 kDa MWCO, Thermo Fisher Scientific, Loughborough, UK). IRDye700DX NHS ester (Rakuten Medical, San Diego, US) further called IR700, was added in a 4.8 molar excess to a 9.3 mg/mL solution of Atezolizumab. The reaction mixture was incubated at 24°C for 1 h in the dark, before purifying using a zeba spin desalting column (40 kDa MWCO, Thermo Fisher Scientific, Loughborough, UK) in PBS. The protein concentration (mM) and the dye/antibody ratio were determined by measuring the UV-Vis absorbance at 278 and 689 nm on a NanoDrop 2000 spectrophotometer (Thermo Fisher Scientific, Loughborough, UK) using 228580 M^-1^cm^-1^ and 165000 M^-1^cm^-1^ as molar extinction coefficients for Atezolizumab and IR700, respectively.

The following formulae were used:

$$Concentration= \frac{A_{280}-(0.123\times A_{689})}{228580}$$

$Ratio=(\frac{A_{689}}{165000})/\frac{A_{280}-(0.123\times A_{689})}{228580}$

An average number of 2.7 dyes per antibody was estimated.

**Preparation of ^89^Zr-DFO-Atezolizumab**

For the conjugation, Atezolizumab solution (Tecentriq®) was buffer exchanged in 0.1 M sodium carbonate pH 9 using a Pierce protein concentrator (50 kDa MWCO). Para-Isothiocyanatobenzyl-desferrioxamine (p-Df-Bz-NCS, 10 mM in DMSO, 2.5 molar eq., CheMatech, Dijon, France) was added to a 6.8 mg/mL Atezolizumab solution. The mixture was incubated at 24°C for 3.5 h before purification using a zeba spin desalting column (40KDa MWCO) in 0.5 M HEPES pH 7. The antibody concentration (mg/mL) was confirmed by measuring the UV absorbance at 278 nm on a NanoDrop 2000 spectrophotometer using a A_0.1%278nm_ of 1.62. The chelate number was determined using the isotopic dilution method described by Deri et al^4^. An average of 1.13±0.03 chelators per protein was estimated. For the radiolabelling, an ^89^Zr-oxalate solution (50-60 MBq, BV CyclotronVU, Amsterdam, the Netherlands) was neutralised with 2.0 M sodium carbonate (Sigma-Aldrich, Gillingham, UK), followed by 0.5 M HEPES pH 7.0 (Sigma-Aldrich). DFO-Atezolizumab was added (250-300 μg) and the mixture was incubated for 1 h at 24°C. At the end of the reaction, a 0.1 M ethylenediaminetetraacetic acid (EDTA, Sigma Aldrich) solution was. The radioconjugate was purified using a zeba spin desalting column (40 kDa MWCO) in 25 mM sodium acetate (Sigma Aldrich). The final protein concentration was determined by UV spectrophotometry at 278 nm.

**Flow Cytometry**

To evaluate the specificity of Atezolizumab, cells were left untreated or pre-blocked with 200-fold excess of unlabelled Atezolizumab (200 nM), followed by incubation with IR700-Atezolizumab (1 nM) at 4°C for 1 h. Cells were washed with PBS and flow cytometry was performed using the FACSymphony A5 Cell Analyser (BD Biosciences, Winnersh, UK) with a total of 10,000 events recorded per sample.

**Tumour and immune cells isolation**

Tissue suspensions were passed through a 70 μm MACSmart Strainer (130-110-916, Miltenyi Biotec, Woking, UK) to obtain a single cell suspension and myelin was removed using the Debris Removal Solution (130-109-398, Miltenyi Biotec, Woking, UK). Red blood cells were lysed using ACK lysis buffer (A1049201, Thermo Fisher Scientific, Loughborough, UK) at room temperature for 5 min. Cells were stained with either live/dead eFluor780 or Zombie UV at 4°C for 10 min, and Fc receptors were blocked with anti-CD16/32 mAb at 4°C for 10 min. Next, cells were incubated with either a T cell or myeloid cell marker panel of fluorescent primary antibodies for 1 h at 4°C. Fluorescent spill-over was compensated with single-colour VersaComp Antibody Capture beads (Beckman Coulter, California, USA) using the FACSDiva software (BD Biosciences, Winnersh, UK). A minimum of 30,000 live events were acquired using the FACSymphony A5 Cell Analyser (BD Biosciences, Winnersh, UK). Data was analysed using FlowJo v9 (BD Biosciences, Winnersh, UK) and gating strategies were implemented for T cell and myeloid cell identification **(Supplementary Figure S4A and S4B)**.

To generate tSNE maps, T cell and myeloid CD45+ populations from 4 independent mice were concatenated into a single dataset and clustered using the tSNE plugin in FlowJo with the following parameters set: Auto (opt-SNE), iterations (1000), perplexity (30), learning rate (1000), exact (vantage point tree), and Barnes-Hut gradient algorithm. Events were subjected to gating strategies previously mentioned (**Supplementary Figure S4A and S4B**) and populations were merged into a single t-SNE map.

Fluorescent primary antibodies were purchased from BioLegend, BD Bioscience or Thermo Fisher Scientific. Antibodies used for T cell population identification included: Fixable Viability Dye eFluor780, CD45 BUV615 (Clone: 30-F11), CD3 BUV737 (Clone: 145-2C11), CD4 BUV496 (Clone: RM4-5), CD8 APC (Clone: 53-6.7), CD25 BV650 (Clone: PC61), PD-1 BV421 (Clone: 29F.1A12), PD-L1 PE (Clone: 10F.9G2). Antibodies used for myeloid cell population identification included: Zombie UV Fixable Viability Dye Kit, CD45 BUV805 (Clone: 30-F11), CD11b APC-Cy7 (Clone: M1/70), CD11c BV785 (Clone: N418), F4/80 BUV661 (Clone: T45-23-42), Gr-1 APC (Clone: RB6-8C5), MHC II PE-Dazzle594 (Clone: M5/114.15.2), PD-1 BV421 (Clone: 29F.1A12), PD-L1 PE (Clone: 10F.9G2).

**Cellular Accumulation of IR700-Atezolizumab**

GL261 cells were seeded in Nunc^TM^ glass bottom petri dishes (Thermo Fisher Scientific, Loughborough, UK) and on the following day treated with 20 ng/ml of recombinant human IFN-γ (Gibco, Thermo Fisher Scientific, Loughborough, UK) or recombinant mouse IFN-γ (Merck KGaA, Darmstadt, Germany) at 37°C for 24 h. The cells were washed with PBS and incubated with IR700-Atezolizumab (1 μM) at either 4°C for 2 h or 37°C for 3 h. Hoechst®33342 (5 μg/mL; Thermo Fisher Scientific, Loughborough, UK) was added for nuclear counterstaining at either 4°C or 37°C for 20 minutes before imaging. Samples were washed twice with PBS post-incubation and images were captured using a Zeiss LSM980 Airyscan 2 confocal microscope (Carl Zeiss Inc, Oberkochen, Germany). Samples were illuminated with 405 and 639 nm lasers and appropriate filters. Data was analysed using the Zen2009 software (Carl Zeiss Inc, Oberkochen, Germany).

**^89^Zr-DFO-Atezolizumab *In Vitro* Studies**

The stability of ^89^Zr-DFO-Atezolizumab, with respect to change in RCP and loss of radioactivity from the radioconjugate, was assessed by incubating the purified product (ca. 3 MBq) in mouse serum (500 μL, Sigma Aldrich, Gillingham, UK) in a thermo shaker (Grant-Bio PHMT, Thermo Fisher Scientific, Loughborough, UK) at 37°C (250 rpm) for up to 4 days. Aliquots were taken at time zero, then at 1, 2, 3 and 4 days and analysed by ITLC using ITLC-SG strips and 0.1 M ammonium acetate and 25 mM EDTA as mobile phase (n=3).

For the specificity of ^89^Zr-DFO-Atezolizumab binding cells pre-treated with media only, 20 ng/ml of recombinant human IFN-γ (Thermo Fisher Scientific, Loughborough, UK) or recombinant mouse IFN-γ (Merck KGaA, Darmstadt, Germany) were incubated with media only or 200-fold molar excess of Atezolizumab (200 nM) for 10 minutes at 4°C. Next, cells were incubated with 1 nM ^89^Zr-DFO-Atezolizumab (2 kBq/well) for 1 h at 4°C. Then, cells were washed with cold-PBS and lysed with RIPA buffer (Thermo Fisher Scientific, Loughborough, UK). The cell-associated and decay-corrected radioactivity was measured on a γ-counter (Wizard^2^ 2480, PerkinElmer, Waltham, UK). The protein concentration in the lysates was determined by the BCA Protein assay (Pierce^TM^ BCA Protein Assay Kit, Thermo Fisher Scientific, Loughborough, UK) and the data was presented as a percentage of incubated dose per mg of the protein lysate (%ID/mg).

**^89^Zr-DFO-Atezolizumab** ***In Vivo* Imaging and *Ex Vivo* Studies**

For PET studies, animals were anaesthetised with isoflurane (3-4% induction, 1-2% maintenance v/v in O_2_ at 1 L/min, ISOFLURIN^®^, Barcelona, Spain) and injected intravenously with ^89^Zr-DFO-Atezolizumab (111 μg Atezolizumab, 0.015-0.018 MBq/μg, 1.72-1.96 MBq/mouse in 100 μL of 0.9% sodium chloride solution). PET/CT scans were acquired 24, 48 and 72 h post-injection using a small-animal PET scanner (Albira PET/SPECT/CT, Bruker, MA, US). Whole body static 15 min PET scans were acquired with a 358 to 664 keV energy window, followed by CT acquisition that was used for scatter and attenuation corrections. PET images were reconstructed using a maximum-likelihood expectation-maximization (MLEN) algorithm (16 interactions) with a voxel size of 0.5×0.5×0.5 mm^3^. High-resolution CT scans were performed with the X-ray tube set-up at a voltage of 45 kV, current of 400 μA, 250 projections (1 s per projection), and a voxel size of 0.5×0.5×0.5 mm^3^. The CT images were reconstructed using a filtered-back-projection (FBP) algorithm. Image analysis was performed using the PMOD software package (PMOD Technologies Ltd., Zurich, Switzerland). Radioactivity uptake in the tumour was quantified by volume-of-interest (VOI) analysis and expressed as the mean (Mean) and the mean of the 50 hottest voxels (Mean_50_) within the VOI. Data was expressed as percentage of the injected dose per gram of tissue (%ID/g) (MBq/g/counts). For biodistribution studies, animals were culled immediately after scanning.

***Ex vivo* Histology and Quantification**

FFPE sections (4 μm) were deparaffinised in xylene (VWR, Pennsylvania, USA) and rehydrated in decreasing concentrations of ethanol (VWR, Pennsylvania, USA).

For H&E, sections were stained with Gill’s haematoxylin (Pioneer Research Chemicals, Colchester, UK) for 1.5 minutes. After washing with H_2_0, sections were stained with eosin (Pioneer Research Chemicals, Colchester, UK) for 2 minutes. Sections were washed with tap water, dehydrated with increasing concentrations of ethanol (VWR, Pennsylvania, USA) and cleared with xylene (VWR, Pennsylvania, USA). Then, coverslips were mounted onto sections using DPX mounting medium (Sigma-Aldrich, Missouri, USA).

For immunohistochemistry, heat-mediated antigen retrieval was performed in citrate antigen retrieval buffer (Vector Laboratories, California, USA) using a microwave at 240 W for 15 min. Endogenous peroxidases were blocked using 0.3% H_2_O_2_ (Sigma-Aldrich, Missouri, USA) at room temperature for 30 min. Non-specific antigen binding was blocked by incubating the slides with 5% rabbit or goat serum (Vector Laboratories, California, USA) at room temperature for 1 h. Sections were incubated with primary antibodies at 4°C overnight in a humidified chamber (**Supplementary Table S1**). Sections were then incubated with biotinylated secondary antibodies, followed by Avidin-Biotin complex from the VECTASTAIN Elite ABC reagent kit (Vector Laboratories, California, USA) for 1 h at room temperature. DAB peroxidase substrate (Agilent, California, USA) was added to sections until staining was visualised. Slides were counterstained with Gill’s haematoxylin (Pioneer Research Chemicals, Colchester, UK) for 1.5 min, followed by dehydration with ethanol (VWR, Pennsylvania, USA) and clearing with xylene (VWR, Pennsylvania, USA). Coverslips were mounted onto sections using DPX mounting medium. Slides were digitally processed using the Nanozoomer-XR slide scanner (Hamamatsu Photonics, Japan). Image analysis and digital scoring of PD-L1 expression was performed using QuPath (Queen’s University, Belfast, Northern Ireland)^6^. Colour deconvolution was performed by setting appropriate haematoxylin and DAB staining vectors. Cell detection was performed with default setup parameters except for Requested pixel size (0.25 μm), Sigma (2 μm), and deselection of Exclude DAB *(membrane staining)*. Single measurement classifiers were set and run for the measurement of *Cell: DAB OD mean* with appropriate thresholding and the mean % PD-L1-positive cells per GL261 tumour was derived from all analysed regions.

**Clinical Methods:**

**Patient selection and study design**

The imaging studies involved eight adult patients with newly diagnosed GBM based on the 2021 WHO Classification of Tumours of the Central Nervous^7^. Inclusion and exclusion criteria are summarized in **Supplementary Table S2**. Eligible patients had GBM located at a sufficient distance from eloquent brain areas, allowing for the possibility of complete resection. So far, all patients had a gross total resection and pre- and post-contrast MRI. ^89^Zr-DFO-Atezolizumab was administered 7 days before surgery. There was no biopsy; however, during surgery, tumour tissue was collected from PET “hot” regions based on PET/MRI images and neuronavigation data. The GBM diagnosis was subsequently confirmed through histopathological and IHC staining. In all enrolled patients so far, there was no discordance between the MRI-established diagnosis of GBM and the post-surgery histological exam. According to the study protocol, patients in this clinical trial can be allocated to one of three study arms based on randomization results: in Arm I, patients receive pembrolizumab as both neoadjuvant and adjuvant treatment; in Arm II, patients receive pembrolizumab only as neoadjuvant treatment; and Arm III, is the control group. All patients are undergoing adjuvant treatment according to the Stupp protocol, which includes radiotherapy plus chemotherapy using temozolomide. In patients presented in this manuscript, three individuals were from the control group and underwent only i.v. administration of ^89^Zr-DFO-Atezolizumab and pre- and post-contrast MRI. Five patients received pembrolizumab only as neoadjuvant treatment, underwent immuno-PET and pre- and post-contrast MRI. Four of these patients underwent MRI and PET scans also 10 weeks post-surgery and one 12 months after surgery.

**^89^Zr-DFO-Atezolizumab clinical production**

DFO-Atezolizumab and ^89^Zr-DFO-Atezolizumab were prepared from commercially available Atezolizumab (Tecentriq, 1200 mg/20 mL, Roche, San Francisco, US) at the Maria Sklodowska-Curie National Research Institute of Oncology in Gliwice, Poland, according to good manufacturing practice (GMP) guidelines and under metal-free conditions. GMP compliant p-Df-Bz-NCS was purchased from DiverChim CDMO (Roissy-en-France, France). The chelate number per antibody was determined by matrix-assisted laser desorption/ionisation time-of-flight mass spectrometry (MALDI Ultraflex TOF/TOF mass spectrometer, Bruker, MA, US). An average of 1.09±0.52 chelators per protein was estimated. GMP compliant ^89^Zr in 1 M oxalic acid was supplied by BV CyclotronVU (Amsterdam, The Netherlands). The radiolabelled product was stable for 72 h. Release specifications are shown in **Supplementary Table S3**. Quality control tests included ITLC to determine the RCP, size-exclusion HPLC analysis for the antibody integrity and assessment of the presence of aggregates, UV spectrophotometry for protein concentration. Additionally, a rapid magnetic beads-based target-binding fraction assay was performed to confirm the binding to PD-L1 as previously described^5^.

**MRI scans**

Images were acquired with MR scanner Siemens Magnetom Prisma 3T using a 20 channels head-neck-spine (HNS) coil. The MRI examination’s protocol included: axial, coronal, sagittal T2-weighted spin-echo imaging, axial pre-contrast T1-weighted spin-echo imaging, axial 3D Fluid Attenuated Inversion

Perfusion-Weighted Imaging (PWI) was performed with the Dynamic Susceptibility Contrast (DSC) method using fast echo planar T2* weighted gradient echo sequence with the following parameters: TR=1430ms, TE=30ms, 4 mm slice thickness, FOV=220mm, gap between slices 20%, matrix size=128.

The DSC perfusion imaging sequence comprised 60 acquisitions and the scan was performed before, during and after the bolus injection. A bolus of gadolinium-based contrast agent in dose 0,1mmol/kg of body weight. At the 10th acquisition the gadolinium-based contrast agent was injected intravenously at the rate of 6 ml/s and followed immediately with the 30 ml saline flush at the same rate. All injections were performed using an automatic power injector. The whole perfusion imaging lasted 1min 33sec with temporal resolution of 1,33 sec. The image processing was performed using commercially available software.

**^89^Zr-DFO-Atezolizumab PET/CT**

Patients were injected with 10 mg of unlabelled Atezolizumab and 37 MBq ± 10% of ^89^Zr-DFO-Atezolizumab (1 mg) via peripheral venous catheter by an infusion pump 7 days prior surgery. The dose was selected based on the previously published protocol^8^. After radioconjugate administration, patients were kept under observation for at least 2  h to identify any infusion-related reactions. PET/CT scans were acquired 48 h and 72 h post-administration using a Siemens Biograph mCT Flow 40-slice PET/CT camera (Siemens Healthineers, Erlangen, Germany). For two subjects this procedure was also performed at 10 weeks post-surgery. During the scan, the patient was in a supine position with arms along the body and the head was placed in a carbon head holder dedicated to brain studies. The whole-body scans were performed from the top of the head up to mid-thigh length (5 min per bed position up to eight bed positions). Low dose CT scans (120 kV, 50 mAs) were acquired for attenuation correction and localization of anatomical structures. Following the whole-body PET, head PET acquisition was run using standard parameters for a brain study and combined with diagnostic CT. In the study performed 48 h post-injection, the acquisition time was set up to 15 min. For scans acquired 72 h post-injection, the time was increased to 20 min to account for the lower activity count rate. All PET images were reconstructed using the corrected TrueX reconstruction algorithm with TOF. The detailed acquisition (CT) and reconstruction (PET) parameters are summarised in **Supplementary Table S4 and S5**. PET acquisitions were fused with a contrast enhanced T1-weighed contrast enhanced (T1*w*CE) MRI sequences. The automatic registration method in the Syngo.via VB40B software (Siemens Healthineers, Erlangen, Germany) was implemented to correct the patient movements. Quantitative data was obtained using Syngo.via VB40B Software and the MM Oncology Module was used for diagnostic examination by physicians. For each PET/CT scan, ROIs were selected as VOI spheres to calculate dose distribution at 48 and 72 h post-injection. Standardised uptake values (SUV) were presented as SUV_max_ normalised to body weight.

**Evaluation of IHC**

FFPE tissue blocks obtained from the patients were cut into 4 μm-thick sections and stained routinely with haematoxylin and eosin (H&E). Initially, the status of isocitrate dehydrogenase 1 (IDH1) mutation was assessed. All tumour samples were confirmed to be IDH1wt and therefore, underwent further analysis. Automated IHC staining for PD-L1, CD4, and CD8 were subsequently performed using the Bench-Mark ULTRA platform (Roche, Basel, Switzerland) and Dako Autostainer Link 48 (Agilent, California, USA) according to manufacturer protocols. Primary antibodies used are stated in **Supplementary Table S6**.

PD-L1 expression was assessed by estimating the intensity of cell membrane staining using a semi-quantitative scale ranging from 0 to 3 (0 = no staining, 1+ = weak staining, 2+ = moderate staining, 3+ = strong staining)^9^. The PD-L1 level was expressed using the Tumour Proportion Score (TPS) and Combined Positive Score (CPS), where TPS is the percentage of PD-L1-positive tumour cells compared to all tumour cells, while CPS is the percentage of PD-L1-positive tumour and inflammatory cells (i.e. lymphocytes and macrophages) compared to all tumour cells^10^.

CD4+ and CD8+ TIL expression was assessed semi-quantitatively by assessing each section using at least five high-power fields (objective ×40 and eyepiece ×10) characterised by the highest TILs abundance. The cells were calculated within the five high-power fields with the most abundant TILs and expressed as a percentage against the tumour tissue background. The mean percentage from those five fields was referred as the percentage of CD4+ and CD8+ TILs for each case^11^.

**Results:**

**S1B.**

**S1A.**

HEPES

Atezolizumab

DFO-Atezolizumab

**Supplementary Figure S1:** **A)** Representative chromatograms of atezolizumab (Tecentriq, 1200mg/20mL, top) and DFO-atezolizumab (bottom). The UV absorbance was measured at the wavelength of 280 nm. Retention times (R_t_) are expressed as minutes : seconds (min : ss). **B)** Representative UV chromatogram (left) and radiochromatogram (right) of purified ^89^Zr-atezolizumab. The UV absorbance was measured at the wavelength of 280 nm.

**GL261_PD-L1KO_**

**2 h**

**37°C**

**S2A.**

**S2B.**

**Supplementary Figure S2:** **A)** Representative confocal immunofluorescence images demonstrating the absence of IR700-Atezolizumab binding in IFN-γ stimulated GL261_PD-L1KO_ cells following incubation with IR700- Atezolizumab at 37°C for 2 hours. Hoechst^®^33342 staining allowed visualisation of cell nuclei. (Scale bar: 5 µM, Magnification: x63). **B)** Binding specificity of IR700-Atezolizumab (1 nM) in murine and human GBM cell lines with and without stimulation with IFN-𝛾 (n=2).

**S3A.**

**PET/CT**

**24 h**

**kBq/cc**

**48 h**

**48 h**

**50**

**300**

**48 h**

**U87-MGvIII**

**S3D.**

**U87-MGvIII**

**H&E**

**PD-L1**

**Ki67**

**S3B.**

**S3C.**

**Supplementary Figure S3:** **A)** Representative PET images of ^89^Zr-DFO-Atezolizumab uptake 24 h and 48 h post-injection in orthotopic U87-MGvIII tumours. T_2_W MRI provides anatomical confirmation of tumours prior to ^89^Zr-DFO-Atezolizumab administration. **B)** PET- image quantification of ^89^Zr-DFO-Atezolizumab brain tumour uptake in non-tumour bearing mice (n=3) or mice bearing orthotopic U87-MGvIII tumours (n=3). **C)** *Ex vivo* biodistribution of non-tumour bearing mice and mice bearing orthotopic U87-MGvIII tumours 72 h after intravenous co-injection of 11 µg ^89^Zr-DFO-Atezolizumab and 100 µg unlabelled Atezolizumab (n=3). **D)** Representative brightfield images of H&E, Ki67, and PD-L1 IHC staining from U87-MGvIII tumour sections (5 µm) (Scale bar: 50 µm, x40 magnification).

**S4A.**

Cells

Single Cells

Live Cells

CD45 BUV615

CD3 BUV737

CD8 APC

SSC-A

FSC-A

CD4 BUV496

CD25 BV650

CD8 APC

PD-L1 PE

PD-1 BV421

PD-L1 PE

PD-1 BV421

PD-L1 PE

PD-1 BV421

CD45+ 70.3%

CD45- 25.9%

CD3+

28.7%

CD4+ CD25+

78.1%

CD4+ CD8-

82.3%

CD4+ CD8+

12.3%

CD4- CD8+

1.64%

CD4- CD8-

3.76%

PD-1+ PD-L1+

62.1%

PD-1- PD-L1+

6.44%

PD-1- PD-L1+

10.3%

PD-1+ PD-L1-

21.2%

PD-1+ PD-L1+

31.6%

PD-1- PD-L1+

0.36%

PD-1- PD-L1+

0%

PD-1+ PD-L1-

68.0%

PD-1+ PD-L1+

76.3%

PD-1- PD-L1+

10.9%

PD-1- PD-L1+

4.0%

PD-1+ PD-L1-

8.9%

CD4+ CD25-

21.3%

**S4B.**

Cells

Single Cells

Live Cells

CD45 BUV805

CD45+ 70.8%

CD45- 21.7%

SSC-A

CD11b APC-Cy7

CD11b+ CD45^high^ 19.7%

CD11b+ CD45^low^ [Microglia] 27.4%

CD11b- CD45+ 37.6%

Gr-1+ [Pan-MDSC]

1.53%

Gr-1-

98.5%

MHCII+

33.3%

MHCII-

66.6%

Gr-1 APC

CD11b APC-Cy7

MHC II PE-Dazzle594

CD11b APC-Cy7

PD-L1 PE

PD-1 BV421

PD-L1 PE

PD-1 BV421

PD-1+ PD-L1+

6.37%

PD-1- PD-L1+

90.4%

PD-1+ PD-L1-

2.35%

PD-1- PD-L1-

0.90%

PD-1+ PD-L1+

3.0%

PD-1- PD-L1+

90.3%

PD-1+ PD-L1-

4.07%

PD-1- PD-L1-2.61%

CD11c BV785

Gr-1 APC

PD-L1 PE

PD-1 BV421

PD-1+ PD-L1+

83.4%

PD-1- PD-L1+

0%

PD-1+ PD-L1-

16.6%

PD-1- PD-L1-

0%

CD11c+ Gr-1+

19.6%

CD11c BV785

F4/80 BUV661

CD11c+ F4/80+

65.6%

CD11c- F4/80+

20.1%

CD11c- F4/80-

4.43%

CD11c- F4/80-

9.82%

CD11c BV785

MHC II PE-Dazzle594

MHC II+

74.8%

PD-1 BV421

PD-L1 PE

PD-1 BV421

PD-L1 PE

PD-1+ PD-L1+

32.7%

PD-1- PD-L1+

62.2%

PD-1+ PD-L1-

0.49%

PD-1- PD-L1-4.63%

PD-1+ PD-L1+

20.8%

PD-1- PD-L1+

69.4%

PD-1+ PD-L1-

1.39%

PD-1- PD-L1-8.33%

CD45 BUV805

PD-L1 PE

SOX2 AF488

**Supplementary Figure S4:** Gating strategy used to define **A)** T cell populations and **B)** myeloid cell populations and their level of PD-1 and PD-L1 expression in orthotopic GL261 tumours.

**S5A.**

**72 h p.i.**

Patient 3 (Control)

*Pre-surgery*

**48 h p.i.**

Patient 3 (Control)

*10 weeks post-surgery*

**72 h p.i**

**H&E**

**PD-L1(SP263)**

**Gliosarcoma**

**GBM**

***TPS: 100; CPS:100***

**PET/T1*w*CE Fusion**

**PET/T1*w*CE Fusion**

**50 μm**

**50 μm**

**50 μm**

**H&E**

**PD-L1(SP263)**

***TPS: 40; CPS:45***

**50 μm**

**T1wCE**

**SUV_max_ = 6.9**

**PET/T1*w*CE Fusion**

**S5B.**

**S5C.**

**S5D.**

**72 h p.i.**

**[Gd-] / PET+**

**[Gd+] / PET-**

**[Gd+] / PET+**

Patient 2 (Control)

*Pre-surgery*

**PET/ T1*w*CE Fusion**

**T1*w*CE**

**PWI (rCBV)**

**Supplementary Figure S5:** Representative axial, coronal, and sagittal **A)** pre-surgery and **C)** 10 weeks post-surgery PET images of patient 3 at 48 h and 72 h post- ^89^Zr-DFO-Atezolizumab injection. Contrast-enhanced T_1_W MRI images fused with PET images provide anatomical visualisation of the tumour mass. **B)** Representative images of H&E and PD-L1 IHC staining of multiple biopsy samples taken from patient 3 at the time of surgery. PD-L1 staining was quantified using the tumour proportion score (TPS) and combined positive score (CPS). **D)** T_1_W MRI (left), T_1_W MRI/PET fusion (middle), and perfusion-weighted imaging (right) of the same slice taken from patient 2 which shows regions heterogeneity of ^89^Zr-DFO-Atezolizumab uptake. The orange boxes highlight either gadolinium positive/negative and/or PET positive/negative regions of interest across corresponding T1*w*CE, PET/T1*w*CE fusion and PWI image slices. White arrows further highlight the region of interest.

**50 μm**

**50 μm**

**PET/T1*w*CE Fusion**

**PET/T1*w*CE Fusion**

**PD-L1(SP263)**

**H&E**

**72 h p.i.**

**S6B.**

Patient 5 (*neo*Pembro)

*10 weeks post-surgery*

**S6A.**

**72 h p.i.**

Patient 5 (*neo*Pembro)

*Pre-surgery*

**S6C.**

**Supplementary Figure S6:** Representative axial, coronal, and sagittal **A)** pre-surgery and **B)** 10 weeks post-surgery PET images of patient 5 at 72 h post-^89^Zr-DFO-Atezolizumab injection. Contrast enhanced T_1_W MRI images fused with PET images provide anatomical visualisation of the tumour mass. **C)** Representative images of H&E and PD-L1 IHC staining of a tumour biopsy sample taken from patient 5 at the time of surgery

**S7A.**

**S7B.**

**Supplementary Figure S7:** SUV_max_ biodistribution of ^89^Zr-DFO-Atezolizumab uptake in patients in the **(A)** control (n=3) and **(B)** neoadjuvant pembrolizumab (n=5) groups taken 48 h and 72 h post-injection.

| **Target** | **Clone** | **Host** | **Catalogue number** | **Company** |
| --- | --- | --- | --- | --- |
| **PD-L1** (Murine) | D5V3B | Rabbit IgG | 64988 | Cell Signalling Technology |
| **PD-L1** (Human) | EPR19759 | Rabbit IgG | ab213524 | Abcam |
| **CD4** (Murine) | EPR19514 | Rabbit IgG | ab183685 | Abcam |
| **CD8** (Murine) | 4SM16 | Rat IgG | 14-0195-82 | Thermo Fisher Scientific |
| **Ki67** (Murine) | SP6 | Rabbit IgG | MA5-14520 | Thermo Fisher Scientific |

**Supplementary Table S1:** Primary antibodies used for preclinical IHC staining.

**T1wCE**

**48 h p.i.**

**T1wCE**

| **Inclusion Criteria** | **Exclusion Criteria** |
| --- | --- |
| 1. 18 years ≤ Age ≤ 70 years 2. Clinically and radiologically (contrast CT, full profile MRI – T1-weighted with or without contrast, T2-weighted, FLAIR, DWI, PWI, MR-spectroscopy) confirmed diagnosis of GBM, localized outside eloquent brain areas. 3. Resectable tumor 4. Fully physically active ≥80 points in Karnofsky performance scale 5. Life expectancy of at least 3 months 6. Adequate organ function (confirmed with blood test results within 1 weeks before enrolment) 7. For women of childbearing potential: agreement to remain abstinent (refrain from heterosexual intercourse) or use double barrier contraceptive methods that result in a failure rate of < 1% per year during the treatment period and for at least 120 days after the last immuno-PET imaging. 8. For men: agreement to remain abstinent (refrain from heterosexual intercourse) or use double barrier contraceptive methods that result in a failure rate of < 1% per year during the treatment period and for at least 120 days after the last immuno-PET imaging. | 1. Any active concomitant malignancy 2. Any previous systemic cancer treatment 3. Hypersensitivity or allergy to any substance with similar action mechanism to Pembrolizumab, Atezolizumab, Temozolomide, other monoclonal antibodies or contrast agents 4. Any active immunosuppressive systemic therapy (except corticosteroids under 12 mg) 5. Any active autoimmune disease or systemic therapy for autoimmune disease within 2 years before enrolment 6. History of any immunodeficiency 7. Active infection 8. Significant cardiovascular disease, such as New York Heart Association cardiac disease ≥ Class III, myocardial infarction within 3 months, coronary artery disease, unstable arrhythmias or unstable angina 9. Active liver disease, hepatitis, HBV or HCV infection 10. History of tuberculosis 11. Any mental disorder that may affect patient’s participation. 12. Any drug or psychoactive substance dependence 13. Treatment with any other investigational agent or participation in another clinical trial with therapeutic intent within 28 days prior to study treatment initiation 14. Major surgical procedure within 4 weeks prior to study enrolment or anticipation of need for a major surgical procedure during the course of the study other than for diagnosis 15. Any live vaccination within 30 days before enrolment 16. Any active immunosuppressive systemic infection including history of human immunodeficiency virus (HIV) infection 17. Body mass index (BMI) ≥ 35 kg/m^2^ 18. Pregnant or lactating 19. Tumour other than glioblastoma grade 4 IDH-wildtype, astrocytoma grade 3 or 4 IDH-mutant identified in post-surgery histopathology. 20. Presence of 1p19q codeletion. |

**Supplementary Table S2:** Inclusion and exclusion criteria for patients enrolled to the trial.

| **Test** | **Method** | **Specification** |
| --- | --- | --- |
| **Appearance** | Visual inspection | Clear, colourless solution |
| **pH** | pH strips (Whatman) | 6.5-7.5 |
| **Filter integrity** | Bubble point test | ≥ 4.0 Bar |
| **Radioactive concentration** | Radioactivity and volume measurements | 1.84-2.12 MB/mL (final formulation) |
| **Protein concentration** | UV-Vis (after final filtration) | 0.30 to 0.58 mg/ml |
| **Purity** | ITLC  SE-HPLC | RCP > 95% |
| **Antibody integrity** | SE-HPLC | ≥ 95% monomer  Aggregates <5% |
| **Immunoreactivity (PD-L1 antigen binding)** | Bead-based assay | >90% |
| **Bacterial endotoxins** | Endosafe PTS kinetic test reader | <2.5 EU/mL |
| **Sterility** | Post-release sterility test | No growth |

**Supplementary Table S3:** Release specification of ^89^Zr-DFO-Atezolizumab.

| **CT** | | | | | | |
| --- | --- | --- | --- | --- | --- | --- |
| Protocol: | kV: | mAs: | Slice: | Colimation: | Pitch: | Kernel: |
| Whole-body | 120 | 50 | 3,0 mm | 16 x 1,2 mm | 0,8 | B19f Low Dose  for ECT  B31f medium smooth+ |
| Head | 120 | 340 | 3,0 mm | 40 x 0,6 mm | 0,55 | H19s Low Dose  for ECT  H31s medium smooth+ |

**Supplementary Table S4:** CT acquisition parameters used in PET/CT studies.

| **PET** | | | | | | | |
| --- | --- | --- | --- | --- | --- | --- | --- |
| Protocol: | Reconstruction: | Parameters: | | | | | |
|  |  | Iterations | Zoom | FWHM[mm] | Subsets | Image size: | Filtr: |
| Whole-body | Corrected TrueX + TOF  (ultra HD PET) | 2 | 1 | 3.0 | 21 | 256 | Gaussian |
|  | Uncorrected Itterative | 2 | 1 | 5.0 | 24 | 200 | Gaussian |
| Head | Corrected TrueX + TOF  (ultra HD PET) | 2 | 2 | 2.0 | 21 | 400 | Gaussian |

**Supplementary Table S5:** PET reconstruction parameters used in PET/CT studies.

| **Antibody / Clone** | **Product no.** | **Producer** | **Format** | **Epitope retrieval** | **Visualisation system** | **Immunostainer** |
| --- | --- | --- | --- | --- | --- | --- |
| CD4 / 4B12 | IR649 | Dako/Agilent | Ready to Use (prediluted) | Dako TRS High pH (3in1) | EnVision Flex+ | Dako Autostainer Link48 |
| CD8 / C8/144B | IR623 | Dako/Agilent | Ready to Use (prediluted) | Dako TRS High pH (3in1) | EnVision Flex | Dako Autostainer Link48 |
| PD-1 / NAT105 | 760-4895 | Ventana/Roche | Ready to Use (prediluted) | Ultra CC1 | ultraView Universal DAB Detection kit | Ventana BenchMark Ultra |
| PD-L1 / SP263 | 790-4905 | Ventana/Roche | Ready to Use (prediluted) | Ultra CC1 | OptiView DAB IHC Detection Kit | Ventana BenchMark Ultra |

**Supplementary Table S6:** List of mAbs used in IHC studies.

|  | **Control** | | | ***neo*Pembrolizumab** | | | | |
| --- | --- | --- | --- | --- | --- | --- | --- | --- |
| **CD4 [%]** | 36.5 | 32 | 2 | 6.7 | 6 | 16 | 24 | 22 |
| **CD8 [%]** | 20 | 29 | 2 | 28.4 | 6 | 12 | 13 | 10 |
| **PD-L1 CPS** | 25.00 | 45.00 | 5.00 | 0.00 | 1.00 | 17.00 | 25 | 45 |
| **SUV_max_**  **(72 h)** | 6.6 | 6.8 | 6.03 | 2.4 | 4.0 | 5.7 | 7.4 | 13.5 |

**Supplementary Table S7:** CD4, CD8, PD-L1 and SUV_max_ quantification.

**References**

**1.**	Pollard SM, Yoshikawa K, Clarke ID, et al. Glioma Stem Cell Lines Expanded in Adherent Culture Have Tumor-Specific Phenotypes and Are Suitable for Chemical and Genetic Screens. *Cell Stem Cell.* 2009; 4(6):568-580.

**2.**	Workman P, Aboagye EO, Balkwill F, et al. Guidelines for the welfare and use of animals in cancer research. *Brit J Cancer.* 2010; 102(11):1555-1577.

**3.**	Percie du Sert N, Hurst V, Ahluwalia A, et al. The ARRIVE guidelines 2.0: updated guidelines for reporting animal research. *BMJ Open Sci.* 2020; 4(1):e100115.

**4.**	Deri MA, Ponnala S, Kozlowski P, et al. p-SCN-Bn-HOPO: A Superior Bifunctional Chelator for 89Zr ImmunoPET. *Bioconjugate Chemistry.* 2015; 26(12):2579-2591.

**5.**	Sharma SK, Lyashchenko SK, Park HA, et al. A rapid bead-based radioligand binding assay for the determination of target-binding fraction and quality control of radiopharmaceuticals. *Nuclear medicine and biology.* 2019; 71:32-38.

**6.**	Bankhead P, Loughrey MB, Fernández JA, et al. QuPath: Open source software for digital pathology image analysis. *Sci Rep-Uk.* 2017; 7.

**7.**	Berger TR, Wen PY, Lang-Orsini M, Chukwueke UN. World Health Organization 2021 Classification of Central Nervous System Tumors and Implications for Therapy for Adult-Type Gliomas: A Review. *JAMA Oncol.* 2022; 8(10):1493-1501.

**8.**	Bensch F, van der Veen EL, Lub-de Hooge MN, et al. Zr-89-atezolizumab imaging as a non-invasive approach to assess clinical response to PD-L1 blockade in cancer. *Nature Medicine.* 2018; 24(12):1852-+.

**9.**	Garber ST, Hashimoto Y, Weathers SP, et al. Immune checkpoint blockade as a potential therapeutic target: surveying CNS malignancies. *Neuro-Oncology.* 2016; 18(10):1357-1366.

**10.**	De Marchi P, Leal LF, da Silva VD, da Silva ECA, de Lima VCC, Reis RM. PD-L1 expression by Tumor Proportion Score (TPS) and Combined Positive Score (CPS) are similar in non-small cell lung cancer (NSCLC). *J Clin Pathol.* 2021; 74(11):735-740.

**11.**	Han S, Zhang C, Li Q, et al. Tumour-infiltrating CD4 and CD8 lymphocytes as predictors of clinical outcome in glioma. *Brit J Cancer.* 2014; 110(10):2560-2568.
